# Overexpression of Rice *Expansin7* (*Osexpa7*) Confers Enhanced Tolerance to Salt Stress in Rice

**DOI:** 10.3390/ijms21020454

**Published:** 2020-01-10

**Authors:** Chuluuntsetseg Jadamba, Kiyoon Kang, Nam-Chon Paek, Soo In Lee, Soo-Cheul Yoo

**Affiliations:** 1 Crop Molecular Breeding Laboratory, Department of Plant Life and Environmental Science, Hankyong National University, Jungangro, Anseong-si, Gyeonggi-do 17579, Korea; chukaj42@gmail.com; 2 Department of Plant Science, Plant Genomics and Breeding Institute, Research Institute of Agriculture and Life Sciences, Seoul National University, Seoul 151-921, Korea; kykang7408@snu.ac.kr (K.K.); ncpaek@snu.ac.kr (N.-C.P.); 3 Department of Agricultural Biotechnology, National Institute of Agricultural Sciences (NAS), RDA, Jeonju 54874, Korea

**Keywords:** *OsEXPA7*, salt tolerance, cell expansion, RNA sequencing, Na^+^ exclusion, root development, auxin, stress

## Abstract

Expansins are key regulators of cell-wall extension and are also involved in the abiotic stress response. In this study, we evaluated the function of *OsEXPA7* involved in salt stress tolerance. Phenotypic analysis showed that *OsEXPA7* overexpression remarkably enhanced tolerance to salt stress. *OsEXPA7* was highly expressed in the shoot apical meristem, root, and the leaf sheath. Promoter activity of OsEXPA7:*GUS* was mainly observed in vascular tissues of roots and leaves. Morphological analysis revealed structural alterations in the root and leaf vasculature of *OsEXPA7* overexpressing (OX) lines. *OsEXPA7* overexpression resulted in decreased sodium ion (Na^+^) and accumulated potassium ion (K^+^) in the leaves and roots. Under salt stress, higher antioxidant activity was also observed in the *OsEXPA7*-OX lines, as indicated by lower reactive oxygen species (ROS) accumulation and increased antioxidant activity, when compared with the wild-type (WT) plants. In addition, transcriptional analysis using RNA-seq and RT-PCR revealed that genes involved in cation exchange, auxin signaling, cell-wall modification, and transcription were differentially expressed between the OX and WT lines. Notably, salt overly sensitive 1, which is a sodium transporter, was highly upregulated in the OX lines. These results suggest that *OsEXPA7* plays an important role in increasing salt stress tolerance by coordinating sodium transport, ROS scavenging, and cell-wall loosening.

## 1. Introduction

Salt stress is one of the most severe environmental stresses that cause plant growth retardation and significant crop loss. More than 20% of agricultural land is affected by high salinity globally and this percentage is expected to increase in the future [1]. Rice, which is a major food crop in several countries, is susceptible to high salinity, especially at the early vegetative and late reproductive stages [2]. Rice plants respond to salt stress at the morphological, anatomical, cellular, and molecular levels [3]. The mechanisms for salt tolerance involve complex stress signaling, and include osmotic adjustment, ion homeostasis, vacuolar compartmentalization of ions, and free radical scavenging [4]. Salinity stress results in the over-accumulation of reactive oxygen species (ROS), such as superoxide radicals (O_2_^−^) and hydrogen peroxide (H_2_O_2_). Over-accumulation of ROS damages various cellular components and macromolecules, including the plasma membrane, nucleic acids, and proteins, which eventually leads to cell death [5]. To cope with this damage, plants induce the expression of genes associated with transcription factors, enzymes, and ion channels [6]. The root is the first organ that responds to salt stress and plays an important role in salinity response [7]. Some salt stress-responsive genes are shown to be mainly induced in the roots [8]. A high concentration of salts, especially sodium chloride (NaCl), dissolved in soil water, induces osmotic and ionic stress resulting in altered K^+^/Na^+^ ratios and high Na^+^ and Cl^–^ ion concentrations, which lead to metabolic changes in the plant [9,10]. Rice plants show tolerance to salt stress via two mechanisms known as ion exclusion and osmotic tolerance [11]. Sodium ions (Na^+^) cause growth inhibition via Na^+^ toxicity when present at concentrations higher than the optimum level (1–10 mM) in the cytosol [12]. Potassium ions (K^+^) are the most abundant cations present in the roots and are essential for plant growth and development [13]. Under saline conditions, Na^+^ competes with K^+^ for being taken up through common transport systems since Na^+^ and K^+^ are physicochemically similar monovalent cations. This leads to high Na^+^ and low K^+^ concentrations in the cytosol. The SOS signaling pathway consists of three major proteins known as SOS1, SOS2, and SOS3. SOS1 encodes a plasma membrane-localized Na^+^/H^+^ antiporter that extrudes Na^+^ out of the cell, which reduces the concentration of cytosolic Na^+^ [14,15]. SOS3 and SOS2, which encode a Ca^2+^ binding protein and a protein kinase, respectively, increase salt stress tolerance by sensing the spike in cytosolic Ca^2+^ under salt stress and activating downstream signaling cascades [16,17]. Cytosolic Na^+^ levels can also be reduced via the Na^+^/H^+^ antiporter, which transports Na^+^ to vacuoles. Two types of H^+^ pumps are present in the vacuolar membrane: vacuolar H^+^-ATPase and vacuolar H^+^-pyrophosphatase, which is also known as V-ATPase and V-PPase, respectively [18,19]. Major tonoplast-localized sodium-hydrogen exchanger (NHX) proteins are essential for active K^+^ uptake at the tonoplast for turgor regulation and stomatal function [20]. 

Expansins are cell wall-loosening proteins involved in many developmental and physiological processes [21], including seed germination [22], root hair and architecture regulation [23,24,25], stem and leaf growth [26,27], stomatal movement [26], and fruit softening [28]. The cell wall-loosening property of expansins is important for plants to respond to major abiotic stresses [29]. The cell wall is the first barrier to environmental stresses and should respond to them quickly and reliably by changing its structure or composition. Several studies have shown that genes expressing expansins are induced by environmental stimuli and enhance plant tolerance to various abiotic stresses. These include genes such as *ZmEXPA1*, *ZmEXPA2*, *ZmEXPA6*, and *ZmEXPA8* in maize [30] and *TaEXPB23* in wheat [31], which respond to a water deficit. *GmEXPLB1* in soybeans [32] respond to waterlogging. *IbEXP1*, *IbEXP2*, and *IbEXPL1* genes are located in sweet-potato [33], *AtEXLA2* in are found in Arabidopsis [34], and *TaEXPB7-B* is located in wheat [35], which responds to heat. Additionally, the *AsEXP1* gene is present in turf grass [36], and *TaEXPA2* [37] and *ZmEXPB6* is located in maize [38], which respond to salt tolerance. In rice, *OsEXPA3* is involved in root system development and is highly induced when responding to salt stress [39]. Furthermore, under submergence conditions, *OsEXPA2* and *OsEXPA7* are upregulated and are likely to be involved in rice coleoptile elongation [40]. *OsEXPA7* has been shown to be accumulated under cold stress [41]. Although expansins have been known to respond to abiotic stress in various species, few studies have examined their function in rice.

In this study, we examined the role of OsEXPA7, under salt stress, in rice. We developed several transgenic *OsEXPA7* lines and investigated the phenotypic response of *OsEXPA7*-overexpressing (OX) lines, including changes in the cellular structure of their root and leaf. Moreover, we performed physiological analyses, such as those of changes in ion distribution and ROS production. We also performed RNA-seq analysis of differentially expressed genes (DEGs), in *OsEXPA7*-OX and WT plants under salt stress conditions. The results of this study suggest that *OsEXPA7* overexpression substantially improves salt stress tolerance in rice by regulating ion homeostasis, ROS scavenging, and cell wall-loosening.

## 2. Results

### 2.1. Production and Analysis of OsEXPA7-OX Rice Plants

**Figure 1:**
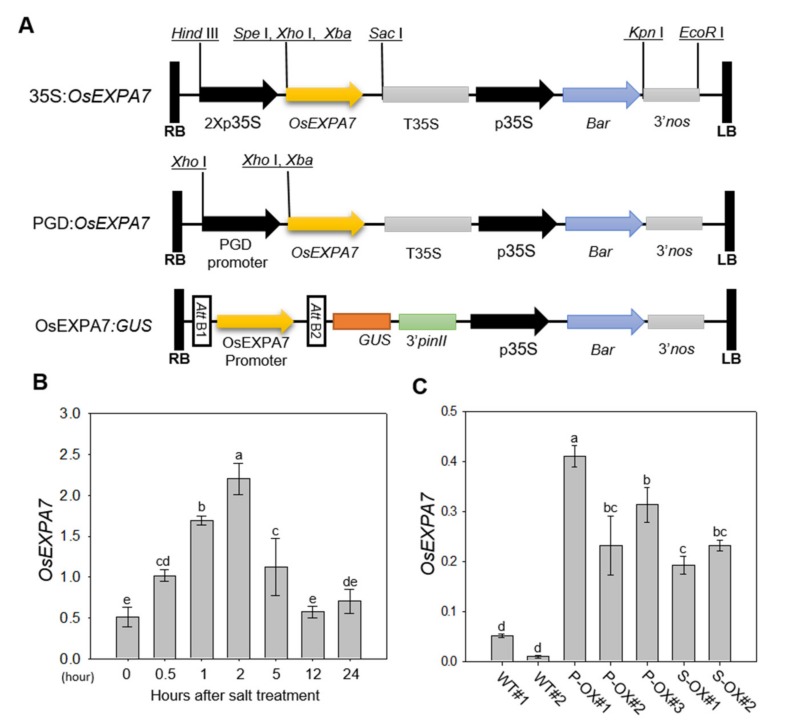
Vector construction and expression analysis of *OsEXPA7* in response to salt stress in wild type and transgenic rice plants. (**A**) Vector construction of transgenic rice lines: 35S:*OsEXPA7*, PGD:*OsEXPA7,* and *OsEXPA7*:*GUS.* RB, right border. LB, left border. 35S, cauliflower mosaic virus 35S promoter. TNOS, nopaline synthase terminator. *Bar*, selection marker. (**B**) Expression pattern of *OsEXPA7* at different time points (0, 0.5, 1, 2, 5, 12, and 24 h), after 150 mM NaCl treatment, determined by qRT-PCR. (**C**) qRT-PCR analysis of *OsEXPA7* expression in two overexpression rice lines, PGD:*OsEXPA7* and 35S:*OsEXPA7.* The housekeeping gene *OsAct11* was used as a control. *OsEXPA7* expression was much higher in the PGD:*OsEXPA7* line than in the WT and 35S:*OsEXPA7* lines. Different letters indicate significant differences according to one-way ANOVA and Duncan’s least significant range test (*p* < 0.05).

Since our previous data showed that *OsEXPA7* expression was highly induced by salt treatment in an RNA-seq analysis (not published), we hypothesized that *OsEXPA7* was involved in the plant response to saline stress. To test this, we prepared a series of transgenic lines including two overexpression (35S:*OsEXPA7* and PGD:*OsEXPA7*) and GUS (*OsEXPA7*:*GUS*) lines. *OsEXPA7* was cloned into pPZP vectors, which contain the promoter for CaMV 35S, phosphogluconate dehydrogenase (PGD), or OsEXPA7, the *PinII* terminator, and the selectable marker *Bar* (Figure 1A). T_2_ lines of *OsEXPA7*-OX transgenic rice plants were generated. The transgenic lines with a single copy number and intergenic insertion of the genes were selected and planted to produce the homozygous T_3_ generation, which were used for functional analysis.

To evaluate the involvement of the *OsEXPA7* gene in the salt stress response, we first analyzed the expression patterns of *OsEXPA7* in WT rice plants after 150 mM of NaCl treatment. Quantitative real-time polymerase chain reaction (qRT-PCR) analysis revealed that the expression level of *OsEXPA7* was induced 0.5 h after salt treatment (HAT) and peaked at 2 HAT (Figure 1B). To select plants highly overexpressing *OsEXPA7*, we next analyzed *OsEXPA7* expression levels in homozygous T_3_ *OsEXPA7*-OX lines, including 35S:*OsEXPA7* (S-OX) and PGD:*OsEXPA7* (P-OX). All overexpression lines exhibited a significantly higher mRNA level than WT plants (Figure 1C). Among them, the P-OX#1 and S-OX#2 lines showed the highest expression for each construct, and were, therefore, used for further analysis.

### 2.2. OsEXPA7 Overexpressing Lines Showed Strong Tolerance to Saline Stress

**Figure 2:**
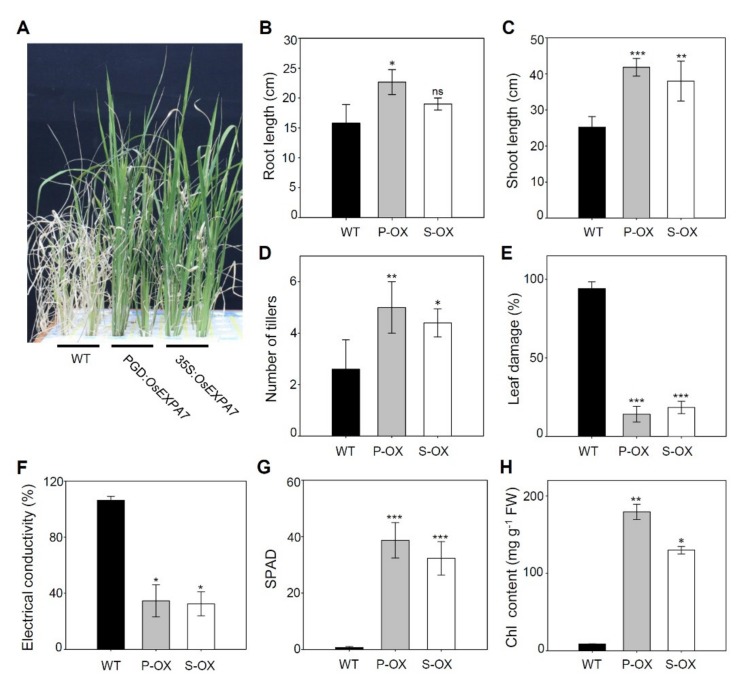
Phenotypic analysis of *OsEXPA7* overexpressing rice plants for salt stress tolerance. Two-week old seedlings grown in a hydroponic solution were treated with 150 mM NaCl for analyzing salt stress response (**A**). Phenotypic analyses were performed by examining root length (**B**), shoot length (**C**), tiller numbers (**D**), leaf damage (**E**), electrical conductivity (**F**), SPAD (**G**), and chlorophyll content (**H**) of the WT plants and the two OX lines (35S:*OsEXPA7* and PGD:*OsEXPA7*). Statistical significance was shown by a *t*-test (*** *p* < 0.001, ** *p* < 0.01, * *p* < 0.05).

To test the phenotypic response of *OsEXPA7*-overexpressing rice plants to saline stress, two transgenic lines (35S:*OsEXPA7* and PGD:*OsEXPA7*) were examined. Two-week-old seedlings, grown hydroponically, were subjected to salt treatment with 150 mM NaCl. Ten days after treatment, all leaves of the WT plant had withered severely and turned white, and the plants nearly died after 16 days (Figure 2A). In contrast, the transgenic lines maintained green leaves with only a few leaf tips being rolled and whitish, and showed almost normal growth. Phenotypic observations showed that both root and shoot lengths were significantly higher in both 35S:*OsEXPA7* and PGD:*OsEXPA7* lines than those in WT plants (Figure 2B,C). The number of tillers had also significantly increased in the lines compared to those in the WT plants (Figure 2D). Plant leaf damage index represents the severity of damage under saline stress. Both the *OsEXPA7*-OX plants (P-OX and S-OX) showed remarkably lower leaf damage than WT plants, which suggests that *OsEXPA7*-OX plants had a higher survival rate compared to that of the WT plants under 150 mM salt stress (Figure 2E). Furthermore, electrical conductivity (EC), which is a parameter for cell damage, was much lower in the OX lines than in the WT plants under salt stress (Figure 2F). One of the main parameters commonly used for evaluating salt stress tolerance is the level of chlorophyll (Chl), which is a photosynthetic pigment. Therefore, we measured Chl content in leaves using an SPAD chlorophyll meter and spectrophotometer. Both OX lines showed higher SPAD values (Figure 2G) and total Chl content compared to the WT plants (Figure 2H). These results indicate that *OsEXPA7* overexpression remarkably increased saline stress tolerance in rice.

### 2.3. OsEXPA7 Was Mainly Expressed in Shoot Apical Meristem (SAM), Root, and Leaf Sheath

**Figure 3:**
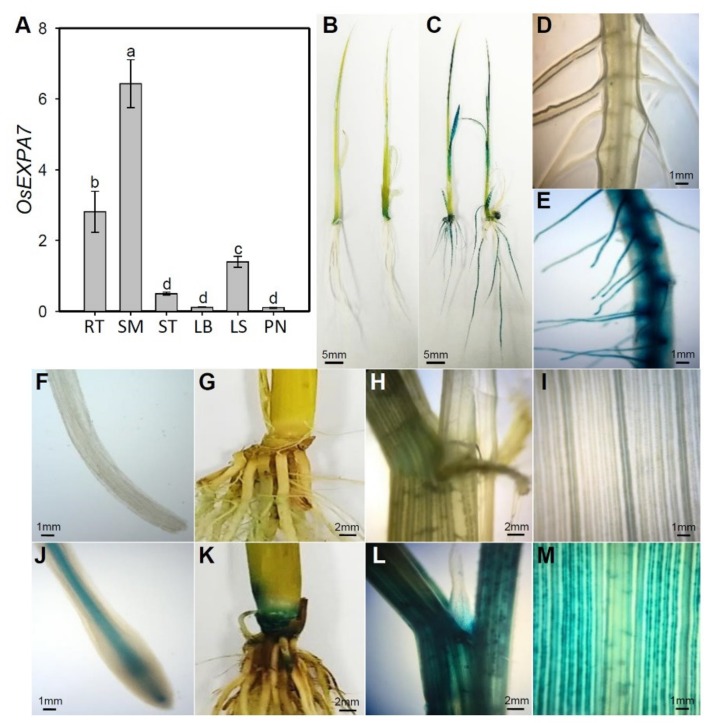
Tissue-specific spatial expression of *OsEXPA7* by histochemical and qRT-PCR analyses. (**A**) qRT-PCR analysis of *OsEXPA7* in various organs. Total RNA was extracted from the root (RT), shoot apical meristem (SM), stem (ST), leaf blade (LB), and leaf sheath (LS) of two-week-old plants and from the panicle (PN) of plants one week after heading. (**B**–**M**) WT and OsEXPA7:*GUS* transgenic rice plants were used for GUS analysis. GUS activity was detected in whole seedlings of WT (**B**) and OsEXPA7:*GUS* (**C**), root hairs of WT (**D**) and OsEXPA7:*GUS* (**E**), root tip of WT (**F**) and OsEXPA7:*GUS* (**J**), SAM of WT (**G**) and OsEXPA7:*GUS* (**K**), leaf sheath of WT (**H**) and OsEXPA7:*GUS* (**L**), and leaf blade of WT (**I**) and OsEXPA7:*GUS* (**M**). Different letters indicate significant differences, according to one-way ANOVA and Duncan’s least significant range test (*p* < 0.05). Photographs shown in (B–M) are representative images.

To elucidate the function of *OsEXPA7* in salt stress tolerance, we evaluated the spatial expression of *OsEXPA7* in various parts of the rice plant. The qRT-PCR analysis showed that *OsEXPA7* was highly expressed in the order of the shoot base (basal regions of the shoot, including apical meristems) > root > leaf sheath (Figure 3A). To further analyze the spatial expression of *OsEXPA7*, we created transgenic rice plants expressing the *GUS* reporter under the control of the OsEXPA7 promoter. Tissue-specific GUS expression was visible as blue staining in the transgenic lines. Intense blue staining was observed in the SAM, root, and leaf sheath of OsEXPA7:*GUS* plants, consistent with the gene expression pattern obtained by qRT-PCR (Figure 3). Particularly, the vasculature, basal region, and hairs of the roots showed intense staining in the transgenic lines, which indicates that these root tissues have strong OsEXPA7 promoter activity (Figure 3C,E,J). In addition, GUS staining was observed in the leaf sheath and shoot base in *OsEXPA7*:*GUS* transgenic rice plants. These results suggest that *OsEXPA7* plays an important role in both the roots and shoots, especially in the vasculature and SAM, where cells are actively dividing and elongating. 

### 2.4. Morphological Changes in Leaves and Roots Due to OsEXPA7 Overexpression in Rice

**Figure 4:**
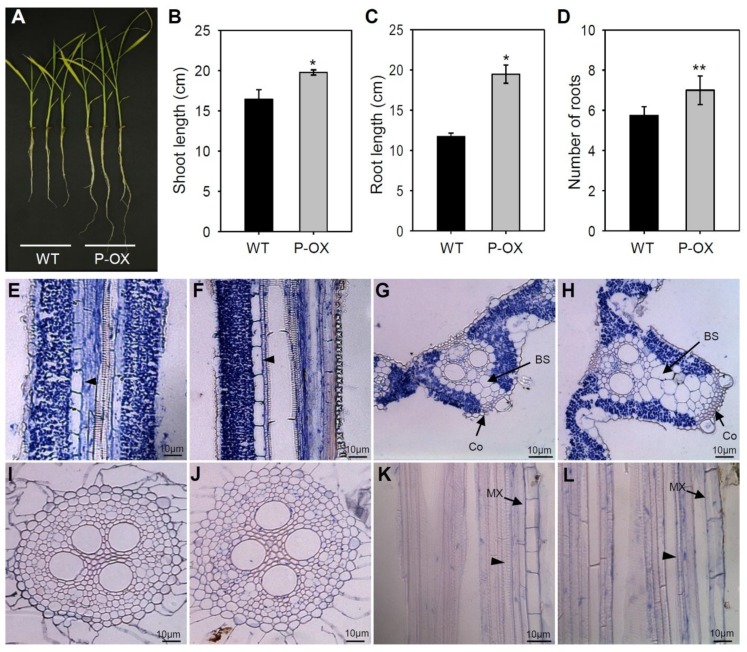
Morphological changes in *OsEXPA7*-OX transgenic plants. (**A**) Phenotypic difference in seven-day-old rice seedlings grown hydroponically under normal nutrition conditions. (**B**) Shoot length, (**C**) root length, (**D**) and number of roots in WT and PGD:*OsEXPA7*. Longitudinal section of leaf blades in WT (**E**) and PGD:*OsEXPA7* plants (**F**) at 40×. Transverse sections of leaf blade in WT (**G**) and PGD:*OsEXPA7* (**H**) at 200×. Transverse sections of primary roots in WT (**I**) and PGD:*OsEXPA7* (**J**). Cell shape and size in WT (**K**) and PGD:*OsEXPA7* (**L**) in longitudinal sections. Arrowheads indicate xylems. Statistical significance was determined by a *t*-test (** *p* < 0.01, * *p* < 0.05). LV, large vein. BS, bundle sheath. Co, collenchyma. MX, metaxylem. Photographs shown in (E–L) are representative images.

Expansins have a cell wall-loosening property that is important for many developmental and physiological processes in plants [21]. To test whether *OsEXPA7* overexpression causes morphological changes in leaves and roots, we evaluated seedling phenotypes of WT and *OsEXPA7*-OX plants grown hydroponically, under normal nutritional conditions. The PGD:*OsEXPA7*#1 (P-OX#1; hereafter *OsEXPA7*-OX) line showed the highest *OsEXPA7* expression among all OX lines and was used for further functional analysis. *OsEXPA7*-OX plants showed a higher biomass both in the shoot and root systems, compared with the WT plants (Figure 4A). Furthermore, we analyzed growth parameters, such as shoot and root lengths and the number of primary roots, in plants growing under control conditions. *OsEXPA7*-OX plants had significantly longer shoot and root lengths and higher root numbers than those of WT plants (Figure 4B–D). To evaluate cell size and shape, we prepared transverse and longitudinal sections of the leaf blades and primary roots of WT and *OsEXPA7*-OX lines, and examined them by optical microscopy. In longitudinal leaf sections, we observed that the metaxylem cells were much longer in *OsEXPA7*-OX plants compared with those in the WT plants (Figure 4E,F). In transverse leaf sections, *OsEXPA7*-OX plants exhibited thicker and larger veins in the leaf blade compared with the WT. Notably, a greater number of larger bundle sheaths (BSs) and collenchyma cells were observed in the large veins of *OsEXPA7*-OX plants compared with their WT counterparts (Figure 4G,H). Low magnification pictures are shown in Supplemental Figure S1A,B. Transverse sections of WT and *OsEXPA7*-OX roots showed the presence of similar sizes and numbers of cells (Figure 4I,J). However, the metaxylem cells in primary roots were longer in *OsEXPA7*-OX plants than those in the WT plants (Figure 4K,L). These results suggest that *OsEXPA7* overexpression promotes an increase in cell size and number in the leaf and elongation of metaxylem cells in the root, which is possibly involved in cell wall loosening, and, thereby, enhances salt tolerance in *OsEXPA7*-OX plants. 

### 2.5. Improved Ion Homeostasis in OsEXPA7-OX Plants Under Salt Stress

**Figure 5:**
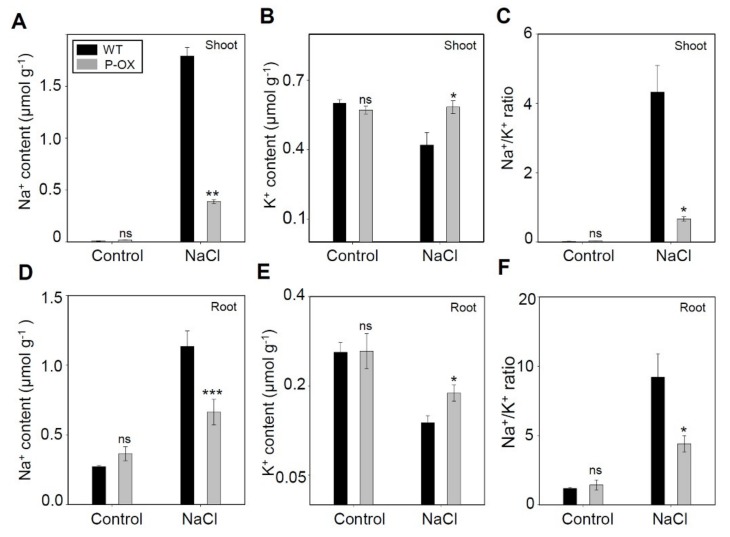
Accumulation of Na^+^ and K^+^ in wild-type and transgenic rice plants under salt stress. Na^+^ content (**A**,**D**), K^+^ content (**B**,**E**), and Na^+^/K^+^ ratios (**C**,**F**) were measured in the shoots and roots of WT and *OsEXPA7*-OX plants grown under 0 and 150 mM NaCl treatment for one week. Ion content was normalized with the dry weight of each sample. All the analyses of ion contents were performed with at least three biological replicates. Asterisks indicate a statistically significant difference, as determined by the Student’s *t*-test (*** *p* < 0.001, ** *p* < 0.01, * *p* < 0.05). ns, not significant.

Na^+^ and K^+^ concentrations are key parameters that determine plant salt stress tolerance, and multiple regulatory pathways regulate them in salt-tolerant plants [42]. Therefore, we measured Na^+^ and K^+^ content in the shoots and roots of *OsEXPA7*-OX and WT plants, using inductively coupled plasma spectrophotometry. Under mock treatment, we observed no substantial difference in either Na^+^ or K^+^ concentration between WT and transgenic plants (Figure 5). However, 7 days after 150 mM NaCl treatment, *OsEXPA7*-OX plants accumulated significantly less Na^+^ in both shoots and roots than WT plants (Figure 5A,B,D,E). Under salinity stress, plants suffer from K^+^ deficiency stemming from the competitive inhibition of K^+^ uptake by Na^+^. Under salt stress, the K^+^ content was maintained at a significantly higher level in both the shoots and the roots of *OsEXPA7*-OX plants compared with those of WT plants (Figure 5B,E). The Na^+^/K^+^ ratio is regarded as an indicator of salt tolerance in plants. Under salt stress, a high Na^+^/K^+^ ratio may impair the selectivity of root cell membranes for ion transport [43]. Our results showed that the Na^+^/K^+^ ratio in the shoot and root tissues of the *OsEXPA7*-OX line was significantly lower than that in the shoots and roots of WT plants (Figure 5C,F). These data demonstrated that *OsEXPA7* inhibits Na^+^ absorption under salt stress, which leads to the maintenance of Na^+^/K^+^ homeostasis and salt tolerance in rice.

### 2.6. Overexpression of OsEXPA7 Improved the Antioxidant Capacity of Transgenic Rice Lines

**Figure 6:**
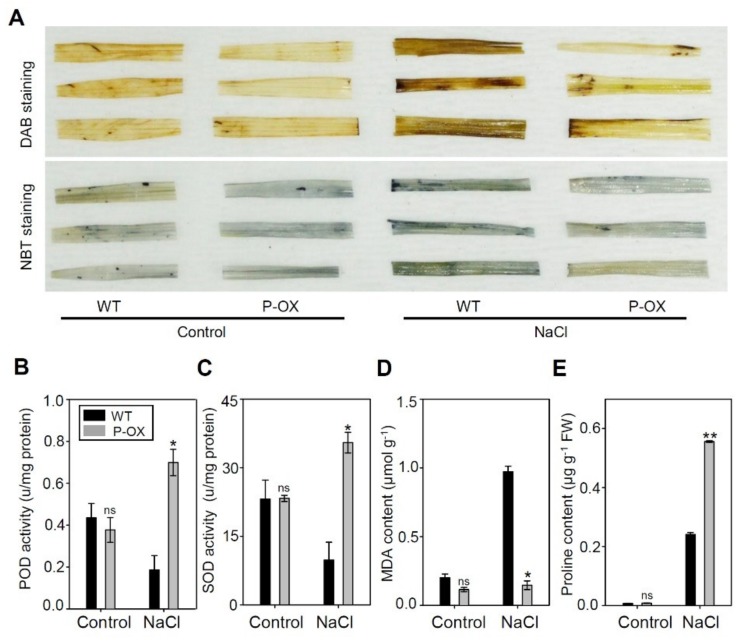
Histochemical staining and antioxidant activity in wild-type and transgenic rice plants under salt stress. (**A**) Detection of H_2_O_2_ and O_2_^−^ by DAB (upper panel) and NBT (bottom panel) staining, respectively, in WT and transgenic seedlings. Seven-day-old plants were grown in normal medium, treated with 0 or 150 mM NaCl for 14 days, and stained as indicated. (**B**,**C**) Antioxidant activity in WT and transgenic leaves. (**D**,**E**) MDA and proline content. POD, peroxidase. SOD, superoxide dismutase. MDA, malondialdehyde. All the experiments were performed with at least three biological replicates. *OsAct11* was used as a control. Asterisks indicate a statistically significant difference, as determined by Student’s *t*-test, (** *p* < 0.01, * *p* < 0.05). Photographs shown in A are representative images. DAB, 3,3′-diaminobenzidine; NBT, nitrotetrazolium blue chloride; MDA, malondialdehyde; ns, not significant.

Rice plants under salt stress exhibit symptoms of oxidative stress, as shown by increased ROS production. Histochemical analysis was, thus, used to investigate the effect of two ROS species including hydrogen peroxide (H_2_O_2_) and superoxide anion (O_2_^−^), using 3,3′-diaminobenzidine (DAB) and nitrotetrazolium blue chloride (NBT) staining, respectively. Under salt stress, the accumulation of brown and blue precipitates (demonstrating DAB and NBT staining, respectively) was much lower in the transgenic lines than in the WT plants. These results suggest that the improved salt stress tolerance of *OsEXPA7*-OX plants may be due to reduced ROS production. To determine whether the reduction in ROS levels was caused by efficient ROS scavenging, we measured the activity of several antioxidant enzymes. Under normal conditions without salt treatment, the activity of peroxidase (POD) and superoxide dismutase (SOD) was nearly identical in both *OsEXPA7*-OX and WT plants. After salt stress treatment, POD and SOD activity significantly increased in *OsEXPA7*-OX plants compared with that in WT plants (Figure 6B,C). Furthermore, the levels of malondialdehyde (MDA), which is a measure of lipid peroxidation, exhibited no difference between *OsEXPA7*-OX and WT under normal conditions. However, the MDA content was significantly lower in *OsEXPA7*-OX plants than in WT plants, which suggests reduced lipid peroxidation in transgenic plants under 150 mM salt stress (Figure 6D). Proline, which is an amino acid, acts as a beneficial solute to increase cellular osmolarity during abiotic stress, by balancing the cytoplasmic osmotic potential and scavenging ROS [44]. The increase in proline accumulation was much higher in *OsEXPA7*-OX plants than in WT plants after salt treatment (Figure 6E).

**Figure 7:**
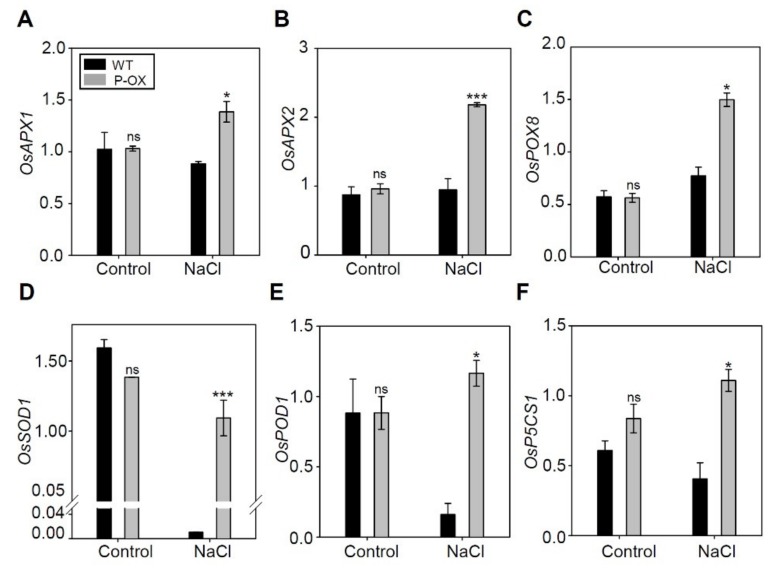
Expression of genes involved in antioxidant activity in the roots of WT and *OsEXPA7*-OX rice plants under salt stress. (**A**–**F**) The expression of genes involved in antioxidant activity was investigated by qRT-PCR. *OsAct11* was used as a control. *OsAPX1* (ascorbate peroxidase 1) (**A**), *OsAPX2* (ascorbate peroxidase 2) (**B**), *OsPOX8* (peroxidase 8) (**C**), *OsSOD1* (superoxide dismutase 1) (**D**), *OsPOD1* (peroxide dismutase 1) (**E**), and *OsP5CS1* (**F**) were tested. qRT-PCR analysis was performed with at least three biological replicates. Asterisks indicate a statistically significant difference, as determined by the Student’s *t*-test (*** *p* < 0.001, * *p* < 0.05). ns, not significant.

To further understand the molecular mechanisms underlying reduced ROS production and increased antioxidant enzyme activity in transgenic rice under salt stress, we investigated the expression levels of several known antioxidant-related genes in rice (Figure 7). The expression levels of *OsAPX1*, *OsAPX2*, *OsPOX8*, *OsSOD1*, and *OsPOD1*, which are involved in ROS scavenging under stress, were almost identical in both WT and transgenic plants under normal conditions without salt treatment. Furthermore, qRT-PCR analysis was carried out for leaf tissues sampled from plants 7 days after salt treatment, and revealed that the expression of all five genes (*OsAPX1*, *OsAPX2*, *OsPOX8*, *OsSOD1*, and *OsPOD1*) was significantly increased in *OsEXPA7*-OX transgenic rice plants compared with WT plants (Figure 7A–E). Furthermore, we measured the expression level of *OsP5CS1*, which is a proline biosynthesis gene, in WT and transgenic plants. *OsP5CS1* expression was significantly upregulated in *OsEXPA7*-OX plants compared to that in WT under salt stress (Figure 7F). These results suggest that *OsEXPA7* overexpression upregulates the expression of major antioxidant-related genes, which reduces ROS production in *OsEXPA7*-OX plants.

### 2.7. DEGs in OsEXPA7-OX and WT Plants

**Figure 8:**
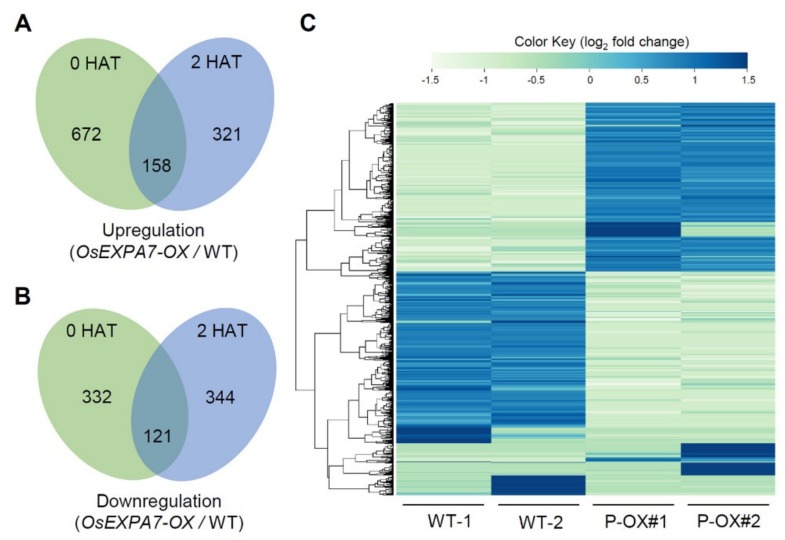
Venn diagram and heatmap of differentially expressed genes (DEGs) under salt stress. (**A**,**B**) Analysis of DEGs in the roots of two-week-old WT and PGD:*OsEXPA7* plants sampled 2 HAT. The Venn diagram shows the overlapping annotations between WT and OX lines. (**C**) Heatmap DEGs.

To obtain genome-wide expression data, transcriptome analysis was conducted using RNA-Seq technology for the roots of two-week-old WT and *OsEXPA7*-OX plants. DEG analysis showed that 672 and 321 genes were upregulated at 0 and 2 HAT, respectively, in *OsEXPA7*-OX plants compared to WT plants (Figure 8A). Among the upregulated genes, 158 genes were common to both 0 and 2 HAT. In contrast, 332 and 344 genes were downregulated at 0 and 2 HAT, respectively, in *OsEXPA7*-OX plants compared with WT plants, while 121 genes were upregulated in both (Figure 8B). The number of upregulated DEGs in *OsEXPA7*-OX plants was around two-fold higher at 0 HAT than at 2 HAT. However, the number of downregulated genes was similar between 0 and 2 HAT. A heatmap was constructed using data for log_2_ fold-change values between WT and *OsEXPA7*-OX plants and log_2_ intensities in two replicates of 944 DEGs, including upregulated and downregulated genes (Figure 8C).

### 2.8. Functional Classification of DEGs Using Gene Ontology (GO) and Kyoto Encyclopedia of Genes and Genomes (KEGG) Pathway Analysis

**Figure 9:**
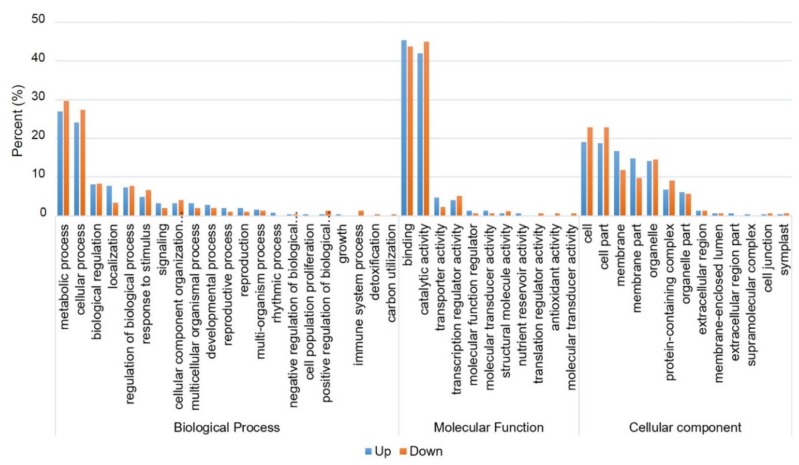
Gene ontology (GO) classification of differentially expressed genes (DEGs) under salt treatment. GO enrichment analysis for upregulated and downregulated genes after salt treatment. The results are divided into three main categories: molecular function, biological process, and cellular component. The Y-axis indicates the percentage of genes in the upregulated and downregulated categories.

To identify the biological processes or pathways that were altered due to salt stress treatment, between WT and *OsEXPA7*-OX plants, GO and KEGG pathway analyses were performed using a false discovery rate (FDR) adjusted with *p* ≤ 0.05 as the cutoff. We annotated genes in three major GO categories including biological process, molecular function, and cellular component. In total, 1478 transcripts were assigned GO terms for DEGs in WT and *OsEXPA7*-OX plants, under salt stress (Table S3). Of these, 545, 326, and 607 transcripts were identified at the biological, molecular, and cellular levels, respectively. These GO terms served as indicators of significantly different functional categories between the roots of PGD:*OsEXPA7* and WT plants, under salt stress. In the biological process category, cellular and metabolic processes were the most highly represented groups, which suggests that extensive metabolic alteration took place in PGD:*OsEXPA7* seedlings compared with WT under salt stress (Figure 9). Within the molecular functional category, transcripts that corresponded to binding and catalytic activities were the most abundant. The cell, cell membrane, and cell organelles were the most abundant groups within the cellular component category.

### 2.9. Genes Involved in Salt Stress Tolerance were Significantly Altered in OsEXPA7-OX Compared to WT

**Figure 10:**
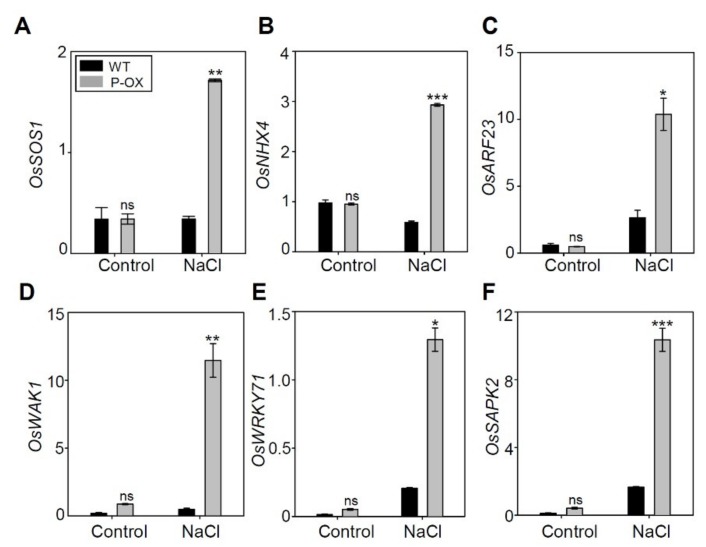
qRT-PCR of candidate genes involved in salt stress tolerance. (**A**–**F**) qRT-PCR amplification of genes from the root tissues of WT and *OsEXPA7*-OX plants, sampled 0 and 2 h after salt treatment. *OsSOS1* (**A**), *OsNHX4* (**B**), *OsARF23* (**C**), *OsWAK1* (**D**), *OsWRKY71* (**E**), and *OsSAPK2* (**F**) were tested. The *OsAct11* expression level was used as a control. qRT-PCR analysis was performed with at least three biological replicates. Asterisks indicate a statistically significant difference, as determined by Student’s *t*-test (*** *p* < 0.001, ** *p* < 0.01, * *p* < 0.05).

Based on the data regarding DEGs between *OsEXPA7*-OX and WT plants under salt treatment, we further analyzed the expression of key genes known to be involved in salt stress tolerance. We selected genes showing greater than two-fold changes. Notably, these genes were related to plasma membrane Na^+^/H^+^ antiporters, WRKY transcription factors, auxin response factors (ARF), and cell wall-associated kinases (WAKs) (Table 1). Na^+^ transporters, namely NHX and SOS1, play major roles in alleviating ionic stress by excluding toxic Na^+^ from the cytosol or preventing Na^+^ transport to photosynthetic organs, and in osmotic stress by modulating the cellular osmotic balance [45]. DEG analysis revealed that *OsSOS1* showed more than two (log_2_ scale)-fold upregulation (2.35) in *OsEXPA7*-OX plants when compared to WT plants under salt stress. However, there was no difference between WT and *OsEXPA7*-OX plants under control conditions. The results were confirmed by qRT-PCR (Figure 10A). *OsNHX4* belongs to the NHX gene family, which plays an important role in salt tolerance by sequestering Na^+^ into the vacuoles of root cells [46]. We evaluated the fold change of the gene from the DEG data. Although *OsNHX4* showed less than a two-fold change in the RNA seq data (Table S4), it was significantly upregulated in qRT-PCR analysis in *OsEXPA7*-OX plants compared with WT (Figure 10B). This indicates that *OsNHX4* is involved in increased salt stress tolerance in *OsEXPA7*-OX plants. The plant hormone, auxin, stimulates cell elongation by increasing cell wall extensibility [47]. Auxin also plays an important role in maintaining the optimal root system architecture that copes with growth reduction caused by water or nutrient shortage. The Oryza sativa auxin response factor 23 (*OsARF23*) is involved in the auxin-actin feedback regulatory loop that is required for auxin-mediated cell growth, especially in root cells [48]. In DEG analysis, *OsARF23* showed a highly increased fold change (25.3-fold upregulation) in *OsEXPA7*-OX plants, which was consistent with the qRT-PCR result (Figure 10C). WAKs have been widely investigated as potential cell wall “sensors,” and they play important roles in cell expansion [49]. RNAi-mediated silencing *OsiWAK1* showed a decrease in plant size due to a reduction in cell size, but not cell number [50]. In DEG analysis, *OsWAK1* showed more than two-fold upregulation (3.93) in *OsEXPA7*-OX plants, which was confirmed by qRT-PCR results (Figure 10D). These results support the finding that *OsWAK1* and *OsARF23* upregulation contributed to the increase in salt stress tolerance in *OsEXPA7*-OX by inducing cell loosening and elongation in the root. WRKY transcription factors are also considered as master regulators of molecular reprogramming to enhance the stress tolerance of plants [51]. Transcription factor *WRKY71* has been shown to help evade salt stress in *Arabidopsis* and enhance cold tolerance in rice [52,53]. DEG analysis revealed that *OsWRKY71* showed upregulation (6.00-fold) in *OsEXPA7*-OX plants under salt stress (Figure 10E), which was confirmed by qRT-PCR results. SNF1-related protein kinase 2 (SnRK2) is a family of kinases that regulates hyperosmotic stress signaling and abscisic acid-dependent development in plants. In rice, the osmotic stress/ABA–activated protein kinase 2 (SAPK2) is the main mediator of ABA signaling [54]. *OsSAPK2* was upregulated more than two-fold (3.30-fold) in our DEG analysis (Table 1). Increased *OsSAPK2* gene expression in *OsEXPA7*-OX plants under salt stress was confirmed by RT-PCR (Figure 10F). Taken together, the results of the DEG analysis revealed that a significant increase in the expression of key genes related to ion homeostasis, cell wall extensibility, and hormone-mediated stress response contributes to enhanced salt stress tolerance in *OsEXPA7*-OX plants under saline stress.

## 3. Discussion

### 3.1. OsEXPA7 Overexpression Improved Salt Stress Tolerance in Rice

Cell wall proteins, including expansins, are believed to play important roles in regulating cell wall extensibility, which, in turn, controls cell enlargement and cell expansion in vivo [55,56]. Expansins are plant cell wall loosening proteins that have been implicated in the control of plant growth via the pH-dependent extension of plant cell walls [57]. Expansins have also been known to be involved in abiotic stress response by cell wall loosening in various species, such as maize [58], soybean [59], *Arabidopsis* [34], and wheat [35]. The involvement of some expansin family genes in the abiotic stress response and plant development has been reported in rice. *OsEXPA3* was shown to be induced in response to salt stress [39], and *OsEXPA7* accumulated under cold stress [41]. *OsEXPA7*, *OsEXPA4*, and *OsEXPA2* have been reported to be upregulated before internode elongation under submergence [40,60]. In addition, it has been reported that *OsEXPA8* regulated leaf size and plant height and improved the root system [61]. Although the involvement of *OsEXPA7* in cold and submergence stress has been reported, its response to salt stress has not been investigated. In this study, we examined the function of *OsEXPA7* in response to salt stress and in the regulation of plant growth at the seedling stage. The accumulation of excessive salt content in the soil results in the inhibition and impairment of crop growth and development. Plant growth inhibition caused by salinity may be associated with cell wall stiffening [62]. Cell expansion is driven by water uptake into the central vacuole [63]. Salt stress restricts cell expansion by affecting water uptake rates, turgor generation, and/or cell wall properties. In this study, we found that *OsEXPA7* expression was highly induced in response to salt stress. Its transcripts peaked 2 h after salt treatment (Figure 1B). *OsEXPA7*-OX transgenic plants showed strong salt stress-tolerant phenotypes with higher chlorophyll content, longer shoots, and lower electrolytic conductivity under 150 mM NaCl stress (Figure 2A–H), which indicates that *OsEXPA7* plays an important role in enhanced tolerance to salinity stress. Spatial expression analysis, qRT-PCR, and GUS staining revealed that *OsEXPA7* is mainly expressed in the stem base, which includes the SAM and root and leaf vasculatures (Figure 3C,D) similarly to other expansins that are mainly expressed in the meristems and growth zones of the root, stem, leaf, and SAM in rice, *Arabidopsis*, and tomato [39,64,65]. Spatial expression patterns show that expansins mainly function in the elongation zones of plants, and, thereby, enables the plants to cope with various abiotic stresses. 

### 3.2. OsEXPA7 Overexpression Increased Cell Size and Enhanced Plant Growth

We hypothesized that the salt tolerance of *OsEXPA7* plants is attributable to cell wall loosening and the subsequent increase in cell size and plant growth. *OsEXPA7*-OX plants showed improved root development (i.e., longer and more primary roots) and significantly higher shoot lengths compared to those of WT plants under normal conditions (Figure 4A–D). At the cellular level, as seen in longitudinal sections, increased length of metaxylem cells was observed in the roots and shoots of *OsEXPA7*-OX plants (Figure 4E,F,K,L). These observations are similar to those reported previously, where *OsEXPA8*-OX plants had longer metaxylem cells in the roots and shoots [61]. Moreover, *OsEXPA3* knockdown by RNAi showed a dramatic decrease in metaxylem cell lengths in the primary root [39]. Since the root system acquires water and nutrients for plant growth and enhances abiotic stress tolerance [66], we reasoned that *OsEXPA7*-OX plants absorbed more water and nutrition through their more developed roots, which leads to enhanced growth and salt tolerance. Previously, it was reported that *GmEXPB2*-OX improved the root system architecture in response to several abiotic stresses [32]. Expansins contributed to cell wall modification and root morphology improvement of the JG11 plant in chickpeas (*Cicer arietinum*) [67]. These results support that *OsEXPA7* is also involved in enhancing growth via acid-mediated cell wall loosening. Furthermore, in transverse leaf sections, *OsEXPA7*-OX showed more numerous and larger vascular BS cells and more collenchyma cells in the large veins of the leaf blade, which resulted in much thicker and bigger large veins than those in WT (Figure 4G,H). However, no apparent difference was observed in the small veins between WT and *OsEXPA7*-OX plants (Figure S1C,D). This is a unique feature of *OsEXPA7*-OX plants that distinguishes them from other expansion mutants. For example, *OsEXPA8*-OX plants showed more cells in many small veins [61]. Vascular bundles act as channels for water and nutrients and for mechanical support in plants [68]. An increase in the number of large veins with bigger and more numerous cells would contribute to enhanced growth in *OsEXPA7*-OX plants. Although *OsEXPA7* overexpression improved shoot and root morphology in the *OsEXPA7*-OX plant seedlings, the yield and yield components were not significantly improved in various transgenic lines, such as 35S:*OsEXPA7,* PGD:*OsEXPA7,* and *OsEXPA7:GUS,* when compared with those in WT plants (Figure S2). This suggests that *OsEXPA7* mainly regulates plant growth at the seedling stage and during stress.

### 3.3. Improvement of Na^+^ and K^+^ Balance in OsEXPA7-OX Plants Under Salt Stress

Many studies have reported that the Na^+^/K^+^ ratio is a reliable indicator of salt stress-tolerance in rice. Lower Na^+^ accumulation was seen in the shoots and roots of *OsEXPA7*-OX plants grown under salt stress (Figure 5A,D). Intracellular Na^+^ is transported out of the cell via the plasma membrane-localized Na^+^/H^+^ antiporter (SOS1) [69] or into the root xylem by the potassium transporter (HKT). Na^+^ can also be compartmentalized into the vacuole via the tonoplast sodium-hydrogen exchanger (NHX) [70]. Our transcriptional analysis revealed that *SOS1* and *NHX4* were significantly upregulated in *OsEXPA7*-OX plants under salt stress (Figure 10A,B). However, *HKT* was not significantly changed, which suggests that cytosolic Na^+^ is excluded primarily by SOS1 and NHX4, rather than by HKT. In *Arabidopsis thaliana*, Na^+^ efflux is likely conducted by the SOS pathway, in which SOS3, known as a calcium-binding protein, interacts with and recruits SOS2, which is a Ser/Thr protein kinase, to the plasma membrane where it activates SOS1 via phosphorylation under salt stress [71,72]. OsSOS1, which is a functional homolog with highly significant sequence similarity to *AtSOS1* [73], also regulates Na^+^ exclusion from the root cells under salt stress [14]. Furthermore, the tonoplast Na^+^/H^+^ antiporters, such as *OsNHX4*, catalyze the sequestration of cytoplasmic Na^+^ to the vacuole [48]. *OsNHX4* was significantly upregulated in the root tissue of *OsEXPA7*-OX plants, which suggests that compartmentalization of Na^+^ into the vacuole contributed to the enhanced tolerance of *OsEXPA7*-OX plants. Moreover, cell elongation via expansin-mediated cell wall-loosening is also expected to reduce the Na^+^ concentration in both the cytoplasm and vacuoles by allowing the absorption of more water than that absorbed by WT plants. Taken together, our results suggest that overexpression of *OsEXPA7* enhanced salt stress tolerance by reducing Na^+^ accumulation and the Na^+^/K^+^ ratio via the SOS and NHX pathways and by cell wall loosening.

### 3.4. Overexpression of OsEXPA7 Improved Oxidative Stress Tolerance

Salt stress causes severe oxidative stress to plants in addition to causing the osmotic stress linked with an increase in phytotoxic ions in the cytosol [74]. High salinity induces the formation of ROS, which can accumulate to cause membrane deterioration, lipid peroxidation, and DNA modification. This leads to metabolic and structural dysfunction [75]. To cope with these oxidative stresses, plants have developed a complex scavenging system involving antioxidants [76]. Low accumulation of superoxide (O_2_^−^), H_2_O_2,_ and lipid peroxidation (measured as MDA content) are the major physiological parameters for oxidative stress tolerance, in addition to enhanced activities of antioxidant enzymes, such as POD and SOD, which catalyze the removal of H_2_O_2_ and O_2_^−^, respectively [75,77]. Moreover, proline accumulation helps to maintain turgor or osmotic balance and reduces ROS levels to within the normal range, which increases tolerance to oxidative and osmotic stress [78]. In this study, *OsEXPA7*-OX plants showed lower ROS accumulation, higher activity of ROS-scavenging enzymes (SOD and POD), a lower MDA concentration, and high proline accumulation (Figure 6). A similar result has been observed in tobacco plants ectopically expressing *TaEXPB23* [79] and *PttEXPA8* [80], which showed drought stress tolerance with lower levels of EC and MDA, higher accumulation of chlorophyll content, and increased SOD activity. Notably, DEG analysis did not identify antioxidant-related genes among the genes showing more than a two-fold change between WT and *OsEXPA7*-OX plants. This is because DEG analysis was performed using RNA extracted from plants treated with 150 mM NaCl for 2 h, which is an insufficient amount of time to accumulate toxic levels of ROS. The ascorbate peroxidase gene *OsAPX1* plays a positive role in chilling tolerance by enhancing H_2_O_2_ scavenging [81], and overexpressing cytosolic copper/zinc superoxide dismutase (*SOD1*) confers abiotic stress tolerance in *Pusa Basmati-1* rice [82]. The expression of several antioxidant-related genes (*OsAPX1*, *OsAPX2*, *OsPOX8*, *OsSOD1,* and *OsPOD1*) was significantly increased in *OsEXPA7*-OX plants 7 days after salt treatment (Figure 7). These results suggest that overexpression of *OsEXPA7* increased antioxidant activity by upregulating antioxidant-related gene expression levels, which alleviated the salt stress-induced oxidative damage.

### 3.5. Possible Mechanisms Underlying Salt Tolerance in OsEXPA7 Overexpressing Rice Plants

The roots are the first organs directly affected by excessive salt in the culture solution. Therefore, RNA-seq was performed using root tissue to identify DEGs between WT and *OsEXPA7*-OX plants under salt stress. *OsARF23* and auxin-responsive *OsGH3-12* were upregulated by more than two (log_2_ scale)-fold (Table 1). Auxin signaling is induced in response to salt stress and mediates a regulatory network that exists between auxin signaling and salt stress [83,84]. *ARF* [85] and *OsGH3* family genes [86] are highly induced under salt stress and play important roles in salinity stress. Higher expression of ARF and auxin-responsive genes suggests that auxin levels might be increased in *OsEXPA7*-OX plants under salt stress compared with the WT. Auxin/indole-3 acetic acid (Aux/IAA), which is a naturally occurring plant hormone of the auxin family, and ARF transcription factors play a critical role in regulating plant development by controlling cell expansion and organ patterning [87]. Auxin activates plasma membrane H^+^-ATPases by upregulating the phosphorylation of plasma membrane H^+^-ATPases, leading to apoplastic acidification [88], which disrupts non-covalent binding between cellulose and hemicelluloses via enhanced expansin activity. This leads to cell wall loosening and elongation [89]. Auxins not only stimulate proton pump activity but also induce gene expression for cation channels, which transport Na^+^ out of the cell, under salt stress [49]. Na^+^ efflux from the plant root is mediated by the activity of the Na^+^/H^+^ antiporter (SOS1) and Na^+^ is compartmentalized into vacuoles by the Na^+^/H^+^ exchanger (NHXs) [90]. Thus, we can infer that the upregulation of *SOS1* and *NHX4* genes in *OsEXPA7*-OX plants may be due to the increased auxin signaling under salt stress. ARF and/or auxin-responsive genes were induced by *OsEXPA7* overexpression, and it seems that a feedback regulatory loop exists where auxin signaling, activated by salt stress, induces *OsEXPA7* expression, which, in turn, increases the expression of genes related to auxin signaling. This enhances salt tolerance. 

In conclusion, our results demonstrated that the overexpression of the α-expansin gene *OsEXPA7* remarkably improves salt stress tolerance. Enhanced salt tolerance may be associated with auxin-induced cell elongation and ion homeostasis as well as enhanced antioxidant competence under salt stress. Overexpressed *OsEXPA7* considerably reduced the accumulation of Na^+^ ion toxicity in the cells and allowed the entry of K^+^ and water into the cytosol, which lowered the Na^+^/K^+^ ratio, possibly via auxin-mediated activation of cation channels and cell elongation. The antioxidative system was also induced more efficiently in *OsEXPA7*-OX, which results in ROS suppression and lower lipid peroxidation under salinity stress when compared to WT. Taken together, our findings suggest that *OsEXPA7* plays an important role in salt stress tolerance at the seedling stage of rice by modulating complex physiological networks.

## 4. Materials and Methods

### 4.1. Plant Materials and Growth Conditions

Salt susceptible rice cultivar Dongjin (*Oryza sativa* L. ssp. japonica) [91] was used as the wild type for physiological and molecular experiments. The *OsEXPA7* (Os03g60720) gene was amplified with the primer set shown in Table S1 to construct various vectors: 35S:*OsEXPA7*, PGD*:OsEXPA7*, and OsEXPA7*:GUS*. Two promoters (35S and PGD) were used to construct overexpression vectors. PGD (phosphogluconate dehydrogenase) is a constitutive promoter that shows high activity at all plant growth stages over three homozygous generations [92,93]. The T_3_ and T_4_ generation plants of homozygous transgenic OX lines were used for all experiments performed in this study. The seeds were sterilized with sodium hypochlorite, germinated at 30 °C for 2 days, and then transferred to a nylon mesh floating on distilled water for 3 days in the hydroponic tank. Thereafter, Yoshida nutrient solution was supplied, which contains macronutrients and micronutrients, with 1 M sulfuric acid (H_2_SO_4_), as described previously [94]. The solutions were replaced weekly and the pH was adjusted to 5.0 each day [95]. Seedlings were grown in a chamber conditioned with 30 °C/25 °C (day/night) and 16 h light/8 h dark at 50%–60% humidity with a light intensity of 20,000–25,000 lux. For the salt stress response experiment, plants were grown in Yoshida nutritional solution supplemented with or without 150 mM NaCl for two to three weeks. For the field test, plant materials were seeded in the greenhouse and transplanted to paddy fields in Anseong, Republic of Korea (37° N latitude) in the last week of May 2019. The plants were cultivated under normal fertilizer conditions (N–P_2_O_5_–K_2_O = 100–80–80 kg/ha) and irrigation.

### 4.2. Morphological Analysis

The shoot length was measured from the shoot base region of the aerial portion to the tip of the longest leaf of the plant, while root length was measured from the shoot base to the tip of the roots. Leaf damage was determined when plants represent a yellow color and witherness in more than 50% of the leaves. For microscopic analysis, fixation, paraffin embedding, and sectioning were conducted as previously described [96]. Fresh leaf and root segments were excised and fixed overnight in a formaldehyde-based fixative (3.7% formaldehyde, 5% acetic acid, and 50% ethanol). Thereafter, leaf and root segments were dehydrated using a series of ethanol concentrations, embedded in paraffin, and sectioned at 8–10 μm thickness with a microtome (Leica RM2125 RTS, Nussloch, Germany). The sections were dried at 40 °C on a hotplate for 8–10 h, deparaffinized with xylene, rehydrated with graded ethanol, and stained with methylene blue for 30 min. Stained sections were examined with optical microscopy at 40× and 200× [97].

### 4.3. Measurement of Electrical Conductivity and Chlorophyll Concentration

The electrolyte leakage ratio (ELR) was measured, as previously described [98]. The third leaves of three-week-old plants were dissected, soaked in a bottle containing 30 mL deionized water, and gently shaken overnight. The initial conductivity of the solution was measured with an EC meter (TDS 6 Plus, Lamotte, Chestertown, MD, USA) (EC1). The total conductivity of the solution was measured (EC2) after the bottles were autoclaved and cooled. ELR was calculated as the ratio of the sample conductivity before autoclaving to that after autoclaving using the following formula: ELR = (EC1/EC2) × 100. The chlorophyll concentration was measured from the middle part of the second youngest leaf in WT and transgenic plants using the SPAD-502 chlorophyll meter (Konica Minolta, Tokyo, Japan). To determine the total chlorophyll content, chlorophyll was extracted from leaf blades of three-week old seedlings with an ice-cold 80% acetone solution. After centrifugation at 10,000× *g* for 15 min at 10 °C, the absorbance of the supernatants was measured at 647 and 663 nm using a UV/VIS spectrophotometer (BioTek Instruments, Winooski, VT, USA). The chlorophyll content was calculated as previously described [99].

### 4.4. Histochemical Assay

For the in situ GUS activity assay, plant materials were histochemically stained in GUS chromogenic solution (10 mM EDTA [pH 8], 50 mM phosphate buffer, 0.1% Triton X-100, 1 mM potassium ferrocyanide [K_4_(Fe[CN]_6_)·3H_2_O], 1 mM potassium ferricyanide [K_3_(Fe[CN]_6_)], 20% methanol, 2 mM X-gluc, and sterile distilled water) and incubated overnight at 37 °C. The ethanol solution was changed twice at 2–3 h intervals. After removing the chlorophyll, the plant materials were rinsed with 70% ethanol and observed with an optical microscope (Lizz, dm optical BL-18T) [100].

### 4.5. Determination of Na^+^ and K^+^ Content

The shoots and roots of three-week old WT and *OsEXPA7*-OX seedlings, grown hydroponically, were harvested. Na^+^ and K^+^ levels were measured according to the methods described previously, with some modifications [101]. The samples were excised from the seedlings, rinsed with deionized H_2_O, and dried in an oven at 70 °C for 2 days. Thereafter, 100 mg of the dried sample was ground with liquid nitrogen, digested with 5 mL of 68% nitric acid and 3 mL of 35.5% hydrogen peroxide for 6 h on a hotplate, and diluted to 25 mL with distilled water. The ion concentrations were analyzed by inductively coupled plasma-optical emission spectrometry (iCAP 7400 Duo, Thermo Fisher Scientific, Waltham, MA, USA).

### 4.6. Determination of ROS and Enzyme Activity 

The formation of hydrogen peroxide (H_2_O_2_) and superoxide anion radicals (O_2_^−^) was detected by DAB and NBT staining, respectively, as described by Bioprotocol [102]. Three biological replicates of rice leaves were first cut into sections and then immersed in 40 mL staining solution (0.1% [*w*/*v*] DAB, pH 6.5, or 0.1% [*w*/*v*] NBT including 10 mM sodium azide and 50 mM potassium phosphate, pH 6.4). Samples were incubated in a growth chamber in the dark overnight. Antioxidant enzyme activities were analyzed according to Giannopolitis & Ries [103]. In addition, 0.5 g of matured fresh leaves were homogenized in an ice-cold mortar in 5 mL of 50 mM sodium phosphate buffer (pH 7.8) containing 1 mM ascorbic acid and 0.5% (*w*/*v*) polyvinylpyrrolidone for 5 min at 4 °C. The homogenate was filtered and centrifuged at 5000× *g* for 15 min. The supernatant was used to determine SOD and POD activities, which were assayed by the NBT and Maehly & Chance methods, respectively [104].

### 4.7. MDA and Proline Content 

In total, 1 g of material was macerated in 5 mL of 0.1% trichloroacetic acid. The homogenate was centrifuged at 10,000× *g* for 5 min. For every 1 mL aliquot of the supernatant, 4 mL of 20% TCA containing 0.5% thiobarbituric acid was added. The mixture was heated at 95 °C for 30 min and then cooled quickly in an ice bath. The resulting mixture was centrifuged at 10,000× *g* for 15 min and the absorbance of the supernatant was measured at 532 nm. Measurements were corrected for nonspecific turbidity by subtracting the absorbance measured at 600 nm. The MDA concentration was calculated by using an extinction coefficient of 155 mM cm^−1^ [105]. The proline content was measured based on the method previously described [106]. Afterward, 0.5 g of dried leaf samples were homogenized with 10 mL of 3% sulfosalicylic acid and filtered through Whatman filter paper #2. Thereafter, 2 mL of filtrate was reacted with 2 mL of ninhydrin and 2 mL of glacial acetic acid in a test tube for 1 h at 100 °C, and the reaction was terminated in an ice bath. The reaction mixture was extracted with 4 mL toluene and mixed vigorously with a test tube stirrer for 15–20 s. The chromophore containing toluene was aspirated from the aqueous phase and warmed to room temperature, and the absorbance was measured at 520 nm using toluene as a blank.

### 4.8. Real-Time PCR Analysis and cDNA Synthesis

For qRT-PCR, total RNAs were extracted by the RNeasy Plant Mini Kit (Qiagen, Hilden, Germany). First-strand cDNA was synthesized from 2 μg of total RNA in a 20 μL volume using M-MLV reverse transcriptase (Promega, Madison, WI, USA) and oligo (dT)_15_ primers, and diluted with 80 μL distilled water. Quantitative PCR was carried out using SYBR Green qPCR SuperMix (Biorad, Hercules, CA, USA). The primers were designed using Primer Express software (Thermo Scientific, Waltham, MA, USA), and the primer sequences used for RT-PCR were listed in Table S2. Each experiment was repeated at least three times. The qPCR amplifications was performed using a Biorad CFX96 (Biorad, Hercules, CA, USA) under the following conditions: 95 °C for 30 s, followed by 39 cycles at 95 °C for 15 s, and 60 °C for 30 s.

### 4.9. RNA Isolation and RNA-Seq Analysis

Total RNA for RNA-Seq was extracted from roots using the RNAeasy Plant Mini Kit (Qiagen, Hilden, Germany). Purified RNA was analyzed using a 2100 Bioanalyzer (Agilent Technologies, Santa Clara, CA, USA) to determine the RNA quantity. Pre-processing of raw data obtained by sequencing was performed for accurate assembly. The Trimmomatic program was used to check the read quality of sequencing data. Adapters and low-quality sequences were removed. After quality trimmings, the pair reads generated through read correction were used for mapping using the HISAT2 program [107]. The read count of the transcript was calculated with StringTie. DEGs were analyzed using DESeq2 [108] based on the read counts of transcripts calculated by StringTie. DEGs exhibiting a two-fold change and false discovery rate (FDR) *p*-values ≤ 0.05 were selected. For functional classification of DEGs, GO was analyzed using BLAST and BLAST2GO (www.blast2go.com), which provides information on gene function. KEGG map analysis was conducted through three-step analyses: BLAST, mapping, and annotation. The KEGG pathway analysis was performed using the hypergeometric test in the R program. Significantly enriched GO terms and KEGG pathways were selected by a threshold FDR (adjusted *p*-value) ≤ 0.05.

### 4.10. Data Analyses

The experiments were designed with a randomized complete block and performed three times. The experimental results were tested by one-way analysis of variance. Unless otherwise noted, the data are means ± standard error (SE). Differences among the treatment means were evaluated using Duncan’s Multiple Range test at *p* < 0.05 with R-studio 3.6.0 (IBM, Boston, MA, USA). Graphs were made using Sigma Plot ver.10.0 (Systat Software, Inc., San Jose, CA, USA).

## Figures and Tables

**Table 1:** List of candidate genes for salt stress tolerance among the differentially expressed genes detected by RNA-sequencing.

Gene Name	Gene ID	Up or Down Ratio (Log2-Fold Change)	Function
*OsSOS1*	LOC_Os12g44360.1	up (2.35)	Sodium/hydrogen exchanger 7, putative, expressed
*OsARF23*	LOC_Os11g32110.3	up (25.30)	auxin response factor, putative, expressed
*OsWAK1*	LOC_Os11g46860.1	up (3.93)	wall-associated receptor kinase 3 precursor
*OsMSRA4.1*	LOC_Os10g41400.2	up (4.64)	response to salt stress, cellular response to oxidative stress
*OsWRKY71*	LOC_Os02g08440.2	up (6.00)	Response to salt stress, response to ethylene, response to abscisic acid
*OsSAPK2*	LOC_Os07g42940.6	up (3.30)	serine/threonine-protein kinase SAPK2
*OsGH3.12*	LOC_Os11g08340.1	up (2.15)	Auxin-responsive Glycoside Hydrolase 3 (GH3) family member

## Supplementary Materials

The following are available online at https://www.mdpi.com/1422-0067/21/2/454/s1. Figure S1: Longitudinal section of leaf blades in WT. Figure S2: Comparison of yield and yield components among three kinds of transgenic plants. Table S1: List of primer sequences used for vector construction. Table S2: List of primer sequences used for RT-PCR analysis. Table S3: Total gene ontology (GO) terms for the DEGs in WT and OsEXAP7-OX plants under salt stress. Table S4: List of candidate genes for salt stress tolerance among the DEGs, detected by RNA-sequencing (lower than log2 scale-fold).

## Abbreviations

CaMV	Cauliflower mosaic virus
PGD	Phosphogluconate dehydrogenase
GUS	b-glucuronidase
WT	Wild type
ROS	Reactive oxygen species
MDA	Malondialdehyde
OX	Overexpression
GO	Gene ontology
LV	Large vein
BS	Bundle sheath
Co	Collenchyma
MX	Metaxylem
ARF	Auxin response factor
SOS1	Salt overly sensitive 1
NHX4	Sodium/hydrogen exchanger 4
PM	Plasma membrane
NaCl	Sodium chloride
DAB	3,3-diaminobenzidine
NBT	Nitro-blue tetrazolium
DEG	Differentially expressed genes
FDR	False discovery rate
qRT-PCR	Quantitative real-time polymerase chain reaction
Aux/IAA	Auxin/indole-3-acetic acid
SAM	Shoot apical meristem
POD	Peroxidase
SOD	Superoxide dismutase
KEGG	Kyoto Encyclopedia of Genes and Genomes
WAK	Wall-associated kinase
ELR	Electrolyte leakage ratio

## References

[1] Munns R. (2005). Genes and salt tolerance: Bringing them together. New Phytol..

[2] Maas E.V. (1986). Salt tolerance of plants. Appl. Agric. Res..

[3] Ashraf M., Akram N.A. (2009). Improving salinity tolerance of plants through conventional breeding and genetic engineering: An analytical comparison. Biotechnol. Adv..

[4] Bohnert H.J., Ayoubi P., Borchert C., Bressan R.A., Burnap R.L., Cushman J.C., Cushman M.A., Deyholos M., Fischer R., Galbraith D.W. (2001). A genomics approach towards salt stress tolerance. Plant Physiol. Biochem..

[5] You J., Chan Z. (2015). ROS Regulation During Abiotic Stress Responses in Crop Plants. Front. Plant Sci..

[6] Xiong L., Schumaker K.S., Zhu J.-K. (2002). Cell Signaling during Cold, Drought, and Salt Stress. Plant Cell.

[7] Galvan-Ampudia C.S., Julkowska M.M., Darwish E., Gandullo J., Korver R.A., Brunoud G., Haring M.A., Munnik T., Vernoux T., Testerink C. (2013). Halotropism Is a Response of Plant Roots to Avoid a Saline Environment. Curr. Biol..

[8] Yan S., Tang Z., Su W., Sun W. (2005). Proteomic analysis of salt stress-responsive proteins in rice root. Proteomics.

[9] Blumwald E. (2000). Sodium transport and salt tolerance in plants. Curr. Opin. Cell Biol..

[10] Louise J., Simon M.-M. (2004). A role for expansins in dehydration and rehydration of the resurrection plant Craterostigma plantagineum. FEBS Lett..

[11] Munns R., Tester M. (2008). Mechanisms of Salinity Tolerance. Annu. Rev. Plant Biol..

[12] Kader M.A., Seidel T., Golldack D., Lindberg S. (2006). Expressions of OsHKT1, OsHKT2, and OsVHA are differentially regulated under NaCl stress in salt-sensitive and salt-tolerant rice (*Oryza sativa* L.) cultivars. J. Exp. Bot..

[13] White P.J., Karley A.J. (2010). Plant Cell Monographs.

[14] Shi H., Ishitani M., Kim C., Zhu J.-K. (2000). The *Arabidopsis thaliana* salt tolerance gene SOS1 encodes a putative Na+/H+ antiporter. Proc. Natl. Acad. Sci. USA.

[15] Gupta B., Huang B. (2014). Mechanism of Salinity Tolerance in Plants: Physiological, Biochemical, and Molecular Characterization. Int. J. Genom..

[16] Kumar K., Kumar M., Kim S.-R., Ryu H., Cho Y.-G. (2013). Insights into genomics of salt stress response in rice. Rice.

[17] Zhu J.-K., Liu J., Xiong L. (1998). Genetic Analysis of Salt Tolerance in *Arabidopsis*: Evidence for a Critical Role of Potassium Nutrition. Plant Cell.

[18] De Lourdes Oliveira Otoch M., Menezes Sobreira A.C., Farias de Aragão M.E., Orellano E.G., da Guia Silva Lima M., Fernandes de Melo D. (2001). Salt modulation of vacuolar H^+^-ATPase and H^+^-Pyrophosphatase activities in Vigna unguiculata. J. Plant Physiol..

[19] Wang B., Lüttge U., Ratajczak R. (2001). Effects of salt treatment and osmotic stress on V-ATPase and V-PPase in leaves of the halophyte Suaeda salsa. J. Exp. Bot..

[20] Barragán V., Leidi E.O., Andrés Z., Rubio L., De Luca A., Fernández J.A., Cubero B., Pardo J.M. (2012). Ion Exchangers NHX1 and NHX2 Mediate Active Potassium Uptake into Vacuoles to Regulate Cell Turgor and Stomatal Function in *Arabidopsis*. Plant Cell.

[21] Sampedro J., Cosgrove D.J. (2005). The expansin superfamily. Genome Biol..

[22] Bae J.M., Kwak M.S., Noh S.A., Oh M.-J., Kim Y.-S., Shin J.S. (2014). Overexpression of sweetpotato expansin cDNA (IbEXP1) increases seed yield in *Arabidopsis*. Transgenic Res..

[23] Zou H., Wenwen Y., Zang G., Kang Z., Zhang Z., Huang J., Wang G. (2015). OsEXPB2, a β-expansin gene, is involved in rice root system architecture. Mol. Breed..

[24] Wang Y., Ma N., Qiu S., Zou H., Zang G., Kang Z., Wang G., Huang J. (2014). Regulation of the α-expansin gene OsEXPA8 expression affects root system architecture in transgenic rice plants. Mol. Breed..

[25] He X., Zeng J., Cao F., Ahmed I.M., Zhang G., Vincze E., Wu F. (2015). HvEXPB7, a novel β-expansin gene revealed by the root hair transcriptome of Tibetan wild barley, improves root hair growth under drought stress. J. Exp. Bot..

[26] Kuluev B.R., Safiullina M.G., Knyazev A.V., Chemeris A.V. (2013). Effect of ectopic expression of NtEXPA5 gene on cell size and growth of organs of transgenic tobacco plants. Russ. J. Dev. Biol..

[27] Goh H.-H., Sloan J., Dorca-Fornell C., Fleming A. (2012). Inducible repression of multiple expansin genes leads to growth suppression during leaf development. Plant Physiol..

[28] Palapol Y., Kunyamee S., Thongkhum M., Ketsa S., Ferguson I.B., van Doorn W.G. (2015). Expression of expansin genes in the pulp and the dehiscence zone of ripening durian (*Durio zibethinus*) fruit. J. Plant Physiol..

[29] Le Gall H., Philippe F., Domon J.-M., Gillet F., Pelloux J., Rayon C. (2015). Cell Wall Metabolism in Response to Abiotic Stress. Plants.

[30] Wu Y., Thorne E.T., Sharp R.E., Cosgrove D.J. (2001). Modification of expansin transcript levels in the maize primary root at low water potentials. Plant Physiol..

[31] Han Y., Chen Y., Yin S., Zhang M., Wang W. (2015). Over-expression of TaEXPB23, a wheat expansin gene, improves oxidative stress tolerance in transgenic tobacco plants. J. Plant Physiol..

[32] Guo W., Zhao J., Li X., Qin L., Yan X., Liao H. (2011). A soybean β-expansin gene GmEXPB2 intrinsically involved in root system architecture responses to abiotic stresses. Plant J..

[33] Noh S.A., Park S.H., Huh G.H., Paek K.-H., Shin J.S., Bae J.M. (2009). Growth retardation and differential regulation of expansin genes in chilling-stressed sweetpotato. Plant Biotechnol. Rep..

[34] Tenhaken R. (2015). Cell wall remodeling under abiotic stress. Front. Plant Sci..

[35] Feng X., Xu Y., Peng L., Yu X., Zhao Q., Feng S., Zhao Z., Li F., Hu B. (2019). TaEXPB7-B, a β-expansin gene involved in low-temperature stress and abscisic acid responses, promotes growth and cold resistance in *Arabidopsis thaliana*. J. Plant Physiol..

[36] Xu J., Tian J., Belanger F.C., Huang B. (2007). Identification and characterization of an expansin gene AsEXP1 associated with heat tolerance in C3Agrostis grass species. J. Exp. Bot..

[37] Chen Y., Han Y., Kong X., Kang H., Ren Y., Wang W. (2017). Ectopic expression of wheat expansin gene TaEXPA2 improved the salt tolerance of transgenic tobacco by regulating Na+/K+ and antioxidant competence. Physiol. Plant..

[38] Geilfus C.-M., Ober D., Eichacker L., Mühling K., Zörb C. (2015). Down regulation of ZmEXPB6 is correlated with salt mediated growth reduction in leaves of Zea mays L.. J. Biol. Chem..

[39] Qiu S., Ma N., Che S., Wang Y., Peng X., Zhang G., Wang G., Huang J. (2014). Repression of OsEXPA3 Expression Leads to Root System Growth Suppression in Rice. Crop Sci..

[40] Lasanthi-Kudahettige R., Magneschi L., Loreti E., Gonzali S., Licausi F., Novi G., Beretta O., Vitulli F., Alpi A., Perata P. (2007). Transcript Profiling of the Anoxic Rice Coleoptile. Plant Physiol..

[41] Bevilacqua C.B., Basu S., Pereira A., Tseng T.-M., Zimmer P.D., Burgos N.R. (2015). Analysis of Stress-Responsive Gene Expression in Cultivated and Weedy Rice Differing in Cold Stress Tolerance. PLoS ONE.

[42] Chen Z., Pottosin I.I., Cuin T.A., Fuglsang A.T., Tester M., Jha D., Zepeda-Jazo I., Zhou M., Palmgren M.G., Newman I.A. (2007). Root Plasma Membrane Transporters controlling K^+^/Na^+^ homeostasis in salt-stressed barley. Plant Physiol..

[43] Ashraf M., Ahmad S. (2000). Influence of sodium chloride on ion accumulation, yield components and fibre characteristics in salt-tolerant and salt-sensitive lines of cotton (*Gossypium hirsutum* L.). Field Crops Res..

[44] Li J., Guo X., Zhang M., Wang X., Zhao Y., Yin Z., Zhang Z., Wang Y., Xiong H., Zhang H. (2018). OsERF71 confers drought tolerance via modulating ABA signaling and proline biosynthesis. Plant Sci..

[45] Yamaguchi T., Hamamoto S., Uozumi N. (2013). Sodium transport system in plant cells. Front. Plant Sci..

[46] Fukuda A., Nakamura A., Hara N., Toki S., Tanaka Y. (2011). Molecular and functional analyses of rice NHX-type Na^+^/H^+^ antiporter genes. Planta.

[47] Majda M., Robert S. (2018). The Role of Auxin in Cell Wall Expansion. Int. J. Mol. Sci..

[48] Li G., Liang W., Zhang X., Ren H., Hu J., Bennett M., Zhang D. (2014). Rice actin-binding protein RMD is a key link in the auxin-actin regulatory loop that controls cell growth. Proc. Natl. Acad. Sci. USA.

[49] Decreux A., Messiaen J. (2005). Wall-associated Kinase WAK1 Interacts with Cell Wall Pectins in a Calcium-induced Conformation. Plant Cell Physiol..

[50] Kanneganti V., Gupta A. (2011). RNAi mediated silencing of a wall associated kinase, 5 OsiWAK1 in Oryza sativa results in impaired root development and sterility due to anther indehiscence. Physiol. Mol. Biol. Plants.

[51] Bakshi M., Oelmüller R. (2014). WRKY transcription factors: Jack of many trades in plants. Plant Signal. Behav..

[52] Yu Y., Wang L., Chen J., Liu Z., Park C.-M., Xiang F. (2017). WRKY71 Acts Antagonistically Against Salt-Delayed Flowering in *Arabidopsis thaliana*. Plant Cell Physiol..

[53] Kumar M., Gho Y.-S., Jung K.-H., Kim S.-R. (2017). Genome-Wide Identification and Analysis of Genes, conserved between japonica and indica Rice Cultivars, that Respond to Low-Temperature Stress at the Vegetative Growth Stage. Front. Plant Sci..

[54] Hu D.-D., Zhang F., Huang L.-Y., Zhuo D.-L., Zhou Y., Shi Y.-Y., Li Z. (2015). Stress-activated Protein Kinase OsSAPK2 Involved in Regulating Resistant Response to *Xanthomonas oryzae* pv. oryzae in Rice. Acta Agron. Sin..

[55] Cosgrove D.J. (1999). Enzymes and Other Agents That Enhance Cell Wall Extensibility. Annu. Rev. Plant Physiol. Plant Mol. Biol..

[56] Taiz L. (1984). Plant Cell Expansion: Regulation of Cell Wall Mechanical Properties. Annu. Rev. Plant Physiol..

[57] Cosgrove D.J. (1998). Cell Wall Loosening by Expansins. Plant Physiol..

[58] Wu Y., Sharp R.E., Durachko D.M., Cosgrove D.J. (1996). Growth maintenance of the maize primary root at low water potentials involves increases in cell-wall extension properties, expansin activity, and wall susceptibility to expansins. Plant Physiol..

[59] Lee D.-K., Ahn J.H., Song S.-K., Choi Y.D., Lee J.S. (2003). Expression of an expansin gene is correlated with root elongation in soybean. Plant Physiol..

[60] Michael A.J. (1996). A cDNA from pea petals with sequence similarity to pollen allergen, cytokinin-induced and genetic tumour-specific genes: Identification of a new family of related sequences. Plant Mol. Biol..

[61] Ma N., Wang Y., Qiu S., Kang Z., Che S., Wang G., Huang J. (2013). Overexpression of OsEXPA8, a Root-Specific Gene, Improves Rice Growth and Root System Architecture by Facilitating Cell Extension. PLoS ONE.

[62] Neumann P.M., Azaizeh H., Leon D. (1994). Hardening of root cell walls: A growth inhibitory response to salinity stress. Plant Cell Environ..

[63] Cosgrove D.J. (1997). Relaxation in a high-stress environment: The molecular bases of extensible cell walls and cell enlargement. Plant Cell.

[64] Cho H.T., Cosgrove D.J. (2000). Altered expression of expansin modulates leaf growth and pedicel abscission in Arabidopsis thaliana. Proc. Natl. Acad. Sci. USA.

[65] Reinhardt D., Wittwer F., Mandel T., Kuhlemeier C. (1998). Localized Upregulation of a New Expansin Gene Predicts the Site of Leaf Formation in the Tomato Meristem. Plant Cell.

[66] Hodge A., Berta G., Doussan C., Merchan F., Crespi M. (2009). Plant root growth, architecture and function. Plant Soil.

[67] Kaashyap M., Ford R., Kudapa H., Jain M., Edwards D., Varshney R., Mantri N. (2018). Differential Regulation of Genes Involved in Root Morphogenesis and Cell Wall Modification is Associated with Salinity Tolerance in Chickpea. Sci. Rep..

[68] Kubo F.C., Yasui Y., Kumamaru T., Sato Y., Hirano H.-Y. (2016). Genetic analysis of rice mutants responsible for narrow leaf phenotype and reduced vein number. Genes Genet. Syst..

[69] Shi H., Quintero F.J., Pardo J.M., Zhu J.-K. (2002). The putative plasma membrane Na^+^/H^+^ antiporter SOS1 controls long-distance Na^+^ transport in plants. Plant Cell.

[70] Apse M.P., Aharon G.S., Snedden W.A., Blumwald E. (1999). Salt Tolerance Conferred by Overexpression of a vacuolar Na^+^/H^+^ antiport in *Arabidopsis*. Science.

[71] Qiu Q.-S., Guo Y., Dietrich M.A., Schumaker K.S., Zhu J.-K. (2002). Regulation of SOS1, a plasma membrane Na^+^/H^+^ exchanger in *Arabidopsis thaliana*, by SOS2 and SOS3. Proc. Natl. Acad. Sci. USA.

[72] Halfter U., Ishitani M., Zhu J.K. (2000). The *Arabidopsis* SOS2 protein kinase physically interacts with and is activated by the calcium-binding protein SOS3. Proc. Natl. Acad. Sci. USA.

[73] Martínez-Atienza J., Jiang X., Garciadeblas B., Mendoza I., Zhu J.-K., Pardo J.M., Quintero F.J. (2007). Conservation of the salt overly sensitive pathway in rice. Plant Physiol..

[74] Hussain S., Shaukat M., Ashraf M., Zhu C., Jin Q., Zhang J. (2019). Climate Change and Agriculture.

[75] Mittler R. (2002). Oxidative stress, antioxidants and stress tolerance. Trends Plant Sci..

[76] Pang C.-H., Wang B.-S. (2008). Progress in Botany.

[77] Ahmad P., Alyemeni M., Abass M., Wijaya L., Alam P., Kumar A., Ashraf M. (2018). Upregulation of antioxidant and glyoxalase systems mitigates NaCl stress in Brassica juncea by supplementation of zinc and calcium. J. Plant Interact..

[78] Hayat S., Hayat Q., Alyemeni M.N., Wani A.S., Pichtel J., Ahmad A. (2012). Role of proline under changing environments: A review. Plant Signal. Behav..

[79] Li F., Xing S., Guo Q., Zhao M., Zhang J., Gao Q., Wang G., Wang W. (2011). Drought tolerance through over-expression of the expansin gene TaEXPB23 in transgenic tobacco. J. Plant Physiol..

[80] Zhang H., Liu H., Yang R., Xu X., Liu X., Xu J. (2019). Over-expression of PttEXPA8 gene showed various resistances to diverse stresses. Int. J. Biol. Macromol..

[81] Sato Y., Masuta Y., Saito K., Murayama S., Ozawa K. (2011). Enhanced chilling tolerance at the booting stage in rice by transgenic overexpression of the ascorbate peroxidase gene, OsAPXa. Plant Cell Rep..

[82] Prashanth S.R., Sadhasivam V., Parida A. (2008). Over expression of cytosolic copper/zinc superoxide dismutase from a mangrove plant *Avicennia marina* in indica Rice var Pusa Basmati-1 confers abiotic stress tolerance. Transgenic Res..

[83] Naser V., Shani E. (2016). Auxin response under osmotic stress. Plant Mol. Biol..

[84] Iqbal N., Umar S., Khan N.A., Khan M.I.R. (2014). A new perspective of phytohormones in salinity tolerance: Regulation of proline metabolism. Environ. Exp. Bot..

[85] Sahi C., Singh A., Kumar K., Blumwald E., Grover A. (2006). Salt stress response in rice: Genetics, molecular biology, and comparative genomics. Funct. Integr. Genom..

[86] Kong W., Zhong H., Deng X., Gautam M., Gong Z., Zhang Y., Zhao G., Liu C., Li Y. (2019). Evolutionary Analysis of GH3 Genes in Six *Oryza* Species/Subspecies and Their Expression under Salinity Stress in *Oryza sativa* ssp. japonica. Plants.

[87] Quint M., Gray W.M. (2006). Auxin signaling. Curr. Opin. Plant Biol..

[88] Ren H., Gray W.M. (2015). SAUR Proteins as Effectors of Hormonal and Environmental Signals in Plant Growth. Mol. Plant.

[89] Perrot-Rechenmann C. (2010). Cellular Responses to Auxin: Division versus Expansion. Cold Spring Harb. Perspect. Biol..

[90] Cho J.-I., Lim H.-M., Siddiqui Z.S., Park S.-H., Kim A.R., Kwon T.-R., Lee S.-K., Park S.-C., Jeong M.-J., Lee G.-S. (2014). Over-expression of PsGPD, a mushroom glyceraldehyde-3-phosphate dehydrogenase gene, enhances salt tolerance in rice plants. Biotechnol. Lett..

[91] Keisham M., Mukherjee S., Bhatla S.C. (2018). Mechanisms of Sodium Transport in Plants-Progresses and Challenges. Int. J. Mol. Sci..

[92] Park S.-H., Yi N., Kim Y.S., Jeong M.-H., Bang S.-W., Choi Y.D., Kim J.-K. (2010). Analysis of five novel putative constitutive gene promoters in transgenic rice plants. J. Exp. Bot..

[93] Park S.-H., Bang S.W., Jeong J.S., Jung H., Redillas M.C.F.R., Kim H.I., Lee K.H., Kim Y.S., Kim J.-K. (2012). Analysis of the APX, PGD1 and R1G1B constitutive gene promoters in various organs over three homozygous generations of transgenic rice plants. Planta.

[94] Gregorio G., Senadhira D., Mendoza R. (1997). Screening Rice for Salinity Tolerance.

[95] Bado S., Forster B.P., Ghanim A.M.A., Jankowicz-Cieslak J., Berthold G., Luxiang L. (2016). Protocol for Screening for Salt Tolerance in Rice. Protocols for Pre-Field Screening of Mutants for Salt Tolerance in Rice, Wheat and Barley.

[96] Periasamy K. (1967). A technique of staining sections of paraffin-embedded plant materials without employing a graded ethanol series. J. R. Microsc. Soc..

[97] Schichnes D., Nemson J., Ruzin S. (2008). Microwave Paraffin Techniques for Botanical Tissues. Microwave Techniques and Protocols.

[98] Wang Y., Jiang J., Zhao X., Liu G., Yang C., Zhan L. (2006). A novel LEA gene from Tamarix androssowii confers drought tolerance in transgenic tobacco. Plant Sci..

[99] Lichtenthaler H.K. (1987). Chlorophylls and carotenoids: Pigments of photosynthetic biomembranes. Methods in Enzymology.

[100] Poosakkannu A., Loganathan A. (2013). Beta glucuronidase activity in early stages of rice seedlings and callus: A comparison with Escherichia coli beta glucuronidase expressed in the transgenic rice. Int. J. Biotechnol. Mol. Biol. Res..

[101] Shen Y., Shen L., Shen Z., Jing W., Ge H., Zhao J., Zhang W. (2015). The potassium transporter OsHAK21 functions in the maintenance of ion homeostasis and tolerance to salt stress in rice. Plant Cell Environ..

[102] Kumar D., Yusuf M.A., Singh P., Sardar M., Sarin N.B. (2014). Histochemical Detection of Superoxide and H_2_O_2_ Accumulation in *Brassica juncea* Seedlings. Bio Protoc..

[103] Giannopolitis C.N., Ries S.K. (1977). Superoxide Dismutases: I. Occurrence in Higher Plants.

[104] Chance B., Maehly A.C. (1955). Assay of catalases and peroxidases. Methods in Enzymology.

[105] Heath R.L., Packer L. (1968). Photoperoxidation in isolated chloroplasts: I. Kinetics and stoichiometry of fatty acid peroxidation. Arch. Biochem. Biophys..

[106] Bates L.S., Waldren R.P., Teare I.D. (1973). Rapid determination of free proline for water-stress studies. Plant Soil.

[107] Pertea M., Kim D., Pertea G.M., Leek J.T., Salzberg S.L. (2016). Transcript-level expression analysis of RNA-seq experiments with HISAT, StringTie and Ballgown. Nat. Protoc..

[108] Huber W., Carey V.J., Gentleman R., Anders S., Carlson M., Carvalho B.S., Bravo H.C., Davis S., Gatto L., Girke T. (2015). Orchestrating high-throughput genomic analysis with Bioconductor. Nat. Methods.

