# Supplementary material for: Overexpression of Rice Expansin7 (Osexpa7) Confers Enhanced Tolerance to Salt Stress in Rice
Source: Int J Mol Sci. 2020 Jan 10;21(2):454. doi: 10.3390/ijms21020454 (PMC7013816; doi:10.3390/ijms21020454)
Supplement: Supplementary file 1 [file ijms-21-00454-s001.pdf]

Supplemental data

Supplemental Table 1. List of primer sequences used for vector construction.

Transgenic line	Primer	Primer sequence (5' -> 3')
**PGD:*OsEXPA7***	XhoI_PGD_F	TTCTCGAG TAGATATGCCGAACATGAC
	XhoI_PGD_R	AACTCGAG TCTGCCTGCAGATAGATG
**35S:*OsEXPA7***	Xba_5UTR_OsEXPA7_F	TCTAGA TCGCACTCACAATGTCG
	SacI_OsEXPA7_R	GAGCTC TCAGACCCGGAAGTTCTTG
**OsEXPA7:GUS**	EXPA7promoter_B1 _F	AAAAAGCAGGCTTACCAAAATTTGCCTATGAT
	EXPA7promoter_B2_R	AGAAAGCTGGGTTGTGAGTGCGAGTGGC

F, forward primer; R, reverse primer.

Supplement Table 2. List of primer sequences used for RT-PCR analysis.

Name	Primer sequence (5' -> 3')
OsACT11-F_qRT	CAGCCACACTGTCCCCATCTA
OsACT11-R_qRT	AGCAAGGTCGAGACGAAGGA
OsEXPA7-F_qRT	GCATCGTCCCCGTCTCCTA
OsEXPA7-R_qRT	GTTCGTGATCAGCACCAGGTT
OsSOD1-F_qRT	CACCACCAATGGTTGCATGT
OsSOD1-R_qRT	TGGCGGGTCTCATCTTCTG
OsAPX1-F_qRT	GGGTTCTGACCACCTAAGG
OsAPX1-R_qRT	CCAGAGAGGGCAACAATGT
OsAPX2-F_qRT	AGCTGCCAAGTGACAAAGC
OsAPX2-R_qRT	CCTCATCCGCAGCATATTTC
OsPOX8-F_qRT	TCTCAGGTGCGCACACGAT
OsPOX8-R_qRT	GCGTCGATGTTGGTCTCGTT
OsPOD1-F_qRT	GTCCGCAAGATCAGCGACTT
OsPOD1-R_qRT	CGGAGTGGAAGGTGACGAT
OsP5CS1-F_qRT	AAGGTGGGCACTGCAGTTGT
OsP5CS1-R_qRT	TCCTTAACCTGCTCGCACAGA
OsNHX4-F_qRT	TCGCGAATCCTGAGGTT
OsNHX4-R_qRT	ACCTGGAAGCCTGCATTG
OsSOS1-F_qRT	TCAGAATGGTGCCAGCTT
OsSOS1-R_qRT	CGTACATGCTCAGCTGCAAT
OsARF23-F_qRT	CCAAATGGTACGCAAAGGAC
OsARF23-R_qRT	TCTAACTCGCTGCTATTGCC
OsWAK1-F_qRT	GAGATGTCAAGCCAGCCAAC
OsWAK1-R_qRT	GCCTAACTTGGAGGACCCAA
OsSAPK2-F_qRT	TGCCGTCAAGTTCATCGA
OsSAPK2-R_qRT	GCCTCAGTGACCTGTGGTT
OsWRKY71-F_qRT	GATGGGTACCAATGGAGGAA
OsWRKY71-R_qRT	GCGGGAGCAAATGAGCAT

F, forward primer; R, reverse primer.

Supplemental Table 3. Total gene ontology (GO) terms for the DEGs in WT and *OsEXAP7*-OX plants under salt stress.

GO TYPE	GO NAME	UP		DOWN	
**Biological Process**	metabolic process	66	26.9%	89	29.7%
	cellular process	59	24.1%	82	27.3%
	biological regulation	20	8.2%	25	8.3%
	Localization	19	7.8%	10	3.3%
	regulation of biological process	18	7.3%	23	7.7%
	response to stimulus	12	4.9%	20	6.7%
	Signaling	8	3.3%	6	2.0%
	cellular component organization or biogenesis	8	3.3%	12	4.0%
	multicellular organismal process	8	3.3%	6	2.0%
	developmental process	7	2.9%	6	2.0%
	reproductive process	5	2.0%	3	1.0%
	Reproduction	5	2.0%	3	1.0%
	multi-organism process	4	1.6%	4	1.3%
	rhythmic process	2	0.8%	0	0.0%
	negative regulation of biological process	1	0.4%	1	0.3%
	cell population proliferation	1	0.4%	0	0.0%
	positive regulation of biological process	1	0.4%	4	1.3%
	Growth	1	0.4%	0	0.0%
	immune system process	0	0.0%	4	1.3%
	Detoxification	0	0.0%	1	0.3%
	carbon utilization	0	0.0%	1	0.3%
**Molecular Function**	Binding	68	45.3%	77	43.8%
	catalytic activity	63	42.0%	79	44.9%
	transporter activity	7	4.7%	4	2.3%
	transcription regulator activity	6	4.0%	9	5.1%
	molecular function regulator	2	1.3%	1	0.6%
	molecular transducer activity	2	1.3%	1	0.6%
	structural molecule activity	1	0.7%	2	1.1%
	nutrient reservoir activity	1	0.7%	0	0.0%
	translation regulator activity	0	0.0%	1	0.6%
	antioxidant activity	0	0.0%	1	0.6%
	molecular transducer activity	0	0.0%	1	0.6%
**Cellular component**	Cell	59	19.0%	68	22.9%
	cell part	58	18.7%	68	22.9%
	Membrane	52	16.8%	35	11.8%
	membrane part	46	14.8%	29	9.8%
	Organelle	44	14.2%	43	14.5%
	protein-containing complex	21	6.8%	27	9.1%
	organelle part	19	6.1%	17	5.7%
	extracellular region	4	1.3%	4	1.3%
	membrane-enclosed lumen	2	0.6%	2	0.7%
	extracellular region part	2	0.6%	0	0.0%

	supramolecular complex	1	0.3%	0	0.0%
	cell junction	1	0.3%	2	0.7%
	Symplast	1	0.3%	2	0.7%
	Total	705	1478	773	

Supplemental Table 4. List of candidate genes for salt stress tolerance among the DEGs, detected by RNA-sequencing (lower than log_2 scale-fold).

Gene name	Gene ID	Up or down ratio (Log2FoldChange)	Function
OsNHX4	LOC_Os06g21360.1	up (0.32)	Response to salt stress;regulation of intracellular pH;potassium ion homeostasis
OsGAP1	LOC_Os02g22130.1	up (0.62)	response to wounding;response to salt stress;abscisic acid-activated signaling pathway
OsCPK13	LOC_Os04g49510.1	up (0.42)	abscisic acid-activated signaling pathway
OsSalT	LOC_Os01g24710.1	up (1.47)	salt stress-induced protein
OsTPP1	LOC_Os02g44230.1	up(0.91)	response to salt stress
OsANN4	LOC_Os05g31760.1	up(1.81)	response to salt stress
OsMAPK5	LOC_Os03g17700.1	up (0.37)	stress-responsive MAPK gene

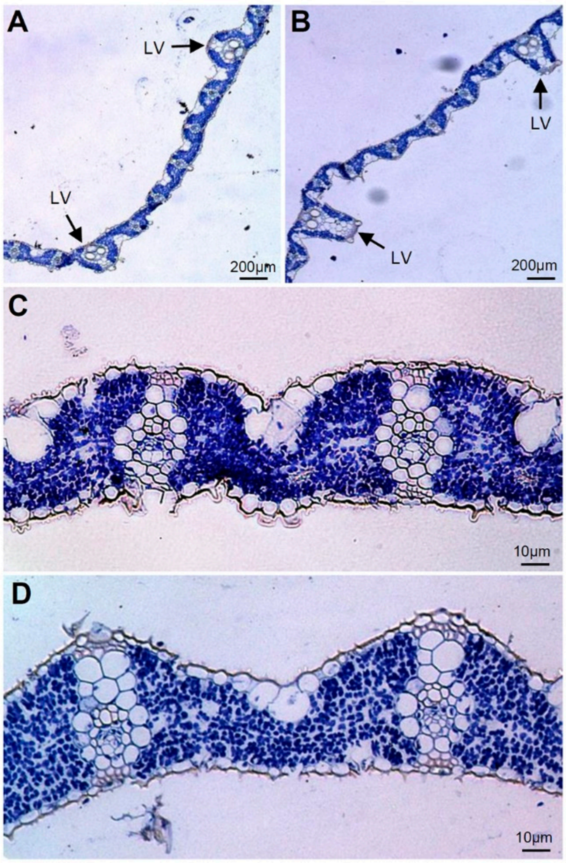

Supplemental Figure 1. Longitudinal section of leaf blades in WT (**A, C**) and *OsEXPA7*-OX plants (**B, D**). Large and small veins in WT **(A)** and *OsEXPA7*-OX **(B)**, and enlarged small veins of WT **(C)** and *OsEXPA7*-OX **(D)**. LV, long vein.

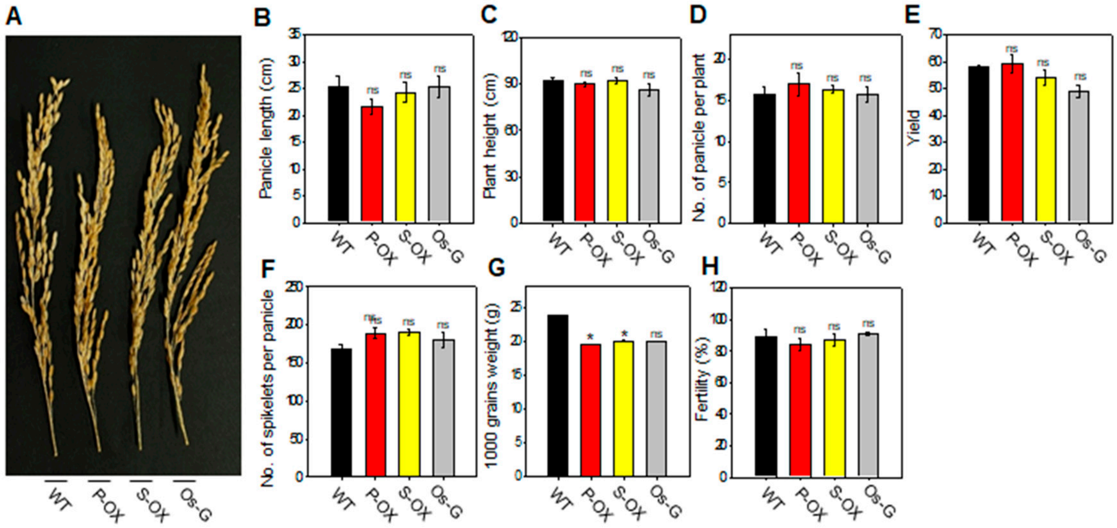

Supplemental Figure 2. Comparison of yield and yield components among three kinds of transgenic plants. Panicle phenotype (A), length of panicle (B), plant height (C), number of panicles per plant (D),

yield (E), number of spikelets per panicle (F), 1000 grains weight (G), and fertility (H). WT, wildtype; P-OX, PGD:*OsEXPA7*, S-OX, 35S:*OsEXPA7*; Os-G, OsEXPA7:*GUS*. Asterisks indicate significant difference examined by Student's t-test. Statistical significance was shown with *p <0.05.
